# The tree balance signature of mass extinction is erased by continued evolution in clades of constrained size with trait-dependent speciation

**DOI:** 10.1371/journal.pone.0179553

**Published:** 2017-06-23

**Authors:** Guan-Dong Yang, Paul-Michael Agapow, Gabriel Yedid

**Affiliations:** 1 Department of Zoology, College of Life Sciences, Nanjing Agricultural University, Nanjing, Jiangsu, China; 2 Data Science Institute, William Penney Laboratory, Imperial College, South Kensington, London, United Kingdom; Western University, CANADA

## Abstract

The kind and duration of phylogenetic topological “signatures” left in the wake of macroevolutionary events remain poorly understood. To this end, we examined a broad range of simulated phylogenies generated using trait-biased, heritable speciation probabilities and mass extinction that could be either random or selective on trait value, but also using background extinction and diversity-dependence to constrain clade sizes. In keeping with prior results, random mass extinction increased imbalance of clades that recovered to pre-extinction size, but was a relatively weak effect. Mass extinction that was selective on trait values tended to produce clades of similar or greater balance compared to random extinction or controls. Allowing evolution to continue past the point of clade-size recovery resulted in erosion and eventual erasure of this signal, with all treatments converging on similar values of imbalance, except for very intense extinction regimes targeted at taxa with high speciation rates. Return to a more balanced state with extended post-extinction evolution was also associated with loss of the previous phylogenetic root in most treatments. These results further demonstrate that while a mass extinction event can produce a recognizable phylogenetic signal, its effects become increasingly obscured the further an evolving clade gets from that event, with any sharp imbalance due to unrelated evolutionary factors.

## Introduction

How the interplay of speciation and extinction has shaped the Tree of Life remains one of the chief unsolved mysteries of evolutionary biology. Processes of origination of new taxa are countered by a steady, low rate of species deaths (background extinction), as well as infrequent but highly destructive episodes of mass extinction. Ideally, tree shape should encode the evolutionary history of the described clade. Metrics of phylogenetic tree shape such as tree “stemminess” (the relative lengths of branches closer to vs. farther from the root) and balance (the degree to which sibling lineages subtend the same number of descendant taxa), might capture lasting “signatures” of past macro-evolutionary processes that affect speciation and extinction [1–3]. However, enthusiasm for this approach should be tempered by the consideration that the same evolutionary processes that produce particular tree shape characteristics can later obscure and eventually erase them [4–6], particularly if the clade is prevented from growing indefinitely [7–11].

Balance has been one of the most studied tree shape metrics [12, 13], usually quantified by a *balance index* (examples in [14–17]), which depends only on tree topology. These indices have been used as tools to both test stochastic models of evolution and departures from them [18–24], and to assess the degree of imbalance of real phylogenies [9, 10, 19, 25–28]. It has been previously asserted that many extant clades (and perhaps the Tree of Life as a whole) are substantially more imbalanced than expected from simple-but-plausible models of diversification [19, 29–39]. Identifying possible causes of high or low clade diversity is therefore important, as well as for the potential to affect other aspects of tree inference [40–42]. In particular, the idea that major macroevolutionary events, especially mass extinctions, can produce long-lasting changes in tree shape has been seductive. While such ideas are quite amenable to exploration with modeling, they have proven difficult to validate due to the relative lack of paleontological data sets with sufficiently high temporal resolution [2, 43, 44]. The most direct demonstration (at least in modeling terms) was shown by Heard and Mooers [45], in the context of clades where speciation rates were controlled by the value of a heritable quantitative trait. Mass extinction that was random produced trees that were more imbalanced compared to their pre-extinction state, as opposed to extinction that was selective on high or low values of the trait, which resulted in more balanced trees vs. the pre-extinction state. This result has become embedded in the literature, having already been used as a conceptual framework for at least one examination using real paleontological data [2]. However, the extent to which a given tree shape property (including balance) will preserve a record of a major evolutionary event depends on a number of factors, including thoroughness of taxon sampling, external constraints on clade size, and how background processes of trait evolution, speciation and extinction have proceeded and affected the clade since the event.

**Fig 1:**
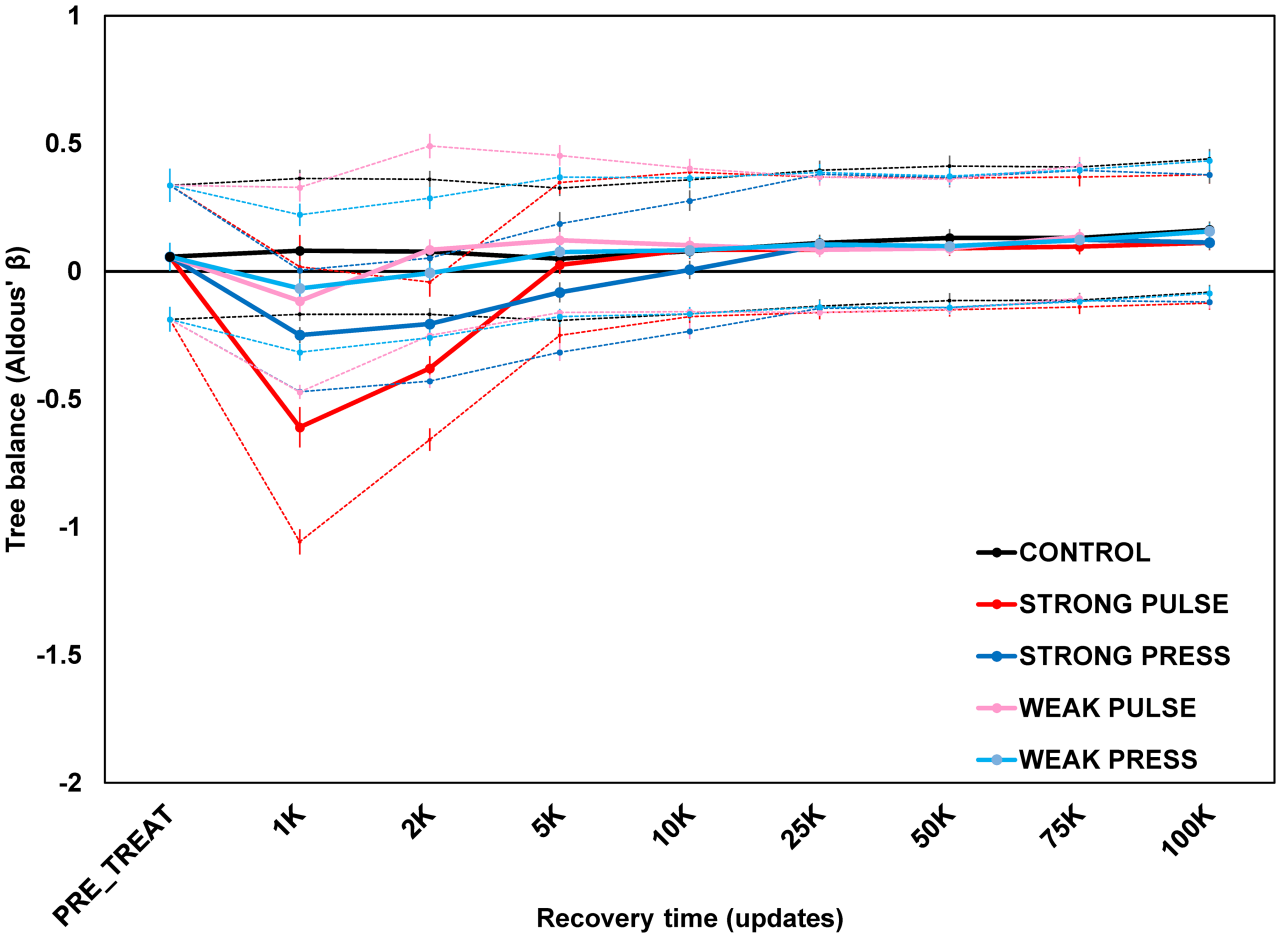
Change in tree balance at select time points after mass extinction episode in communities of avida digital organisms. Data previously unpublished from Yedid et al. (2012). Mass extinction treatments were applied randomly and instantaneously (pulse) or by massive environmental change over a period of time (press), at strong and weak intensities. The y-axis is Aldous’ β [β_A_, 16], a measure of tree balance applicable to non-dichotomous trees; a Yule expectation is around zero, while more negative values indicate trees more imbalanced than this expectation. Data points are averages of 100 replicates ± 2 standard errors. Solid traces are maximum likelihood estimates of β_A_, dashed traces are 95% confidence intervals around the calculated β_A_ estimates. β_A_ values (with confidence intervals) were determined using a customized version of the *maxlik*.*betasplit* function in the R package **apTreeshape** (courtesy M. Blum).

The present study was motivated by previous work [6] highlighting several of the aforementioned issues. We investigated the effects of random and selective mass extinctions on tree stemminess using digital evolution, an individual-based model system very different from the branching process (or birth-death) models usually employed to investigate macroevolution-related questions. With branching process models, the phylogeny itself, and parameters that affect its properties, are the objects of concern. Digital evolution, by contrast, focuses on evolving populations of simulated individual organisms. Each system has particular strengths and drawbacks. Branching process models still provide the most direct way of addressing issues where detailed manipulation of tree properties is needed, but fail to address ecology and interactions among individuals and clades. Digital evolution permits detailed manipulation of individuals, populations, and even ecology. However, the traits that influence probabilities of speciation and extinction are not modeled explicitly as in a branching process and so cannot be manipulated directly, rendering the phylogeny an epiphenomenon of the evolving population. In our previous model [6], mass extinction could be triggered either by instantaneous random culls of a population (pulse extinctions) or by massive environmental changes (press extinctions), and to different degrees of intensity (strong vs. weak). We found that depending on the metric used, different signatures of mass extinction might be retained over the short, but not long term of the recovery, irrespective of treatment. That study did not include results on tree balance: while investigated, the findings failed to confirm the results of Heard and Mooers [45]. Rather, as with one metric of tree stemminess, tree balance showed short-term, but not long-term, differences between different mass extinction treatments (Fig 1), regardless of type or intensity. In particular, tree balance was often indistinguishable from the expectation of a Yule or Equal-Rate Markov (ERM) process (the most common null model of stochastic evolutionary tree growth [32, 46, 47]), with all treatments eventually converging on ERM-like values and only occasional occurrence of significantly imbalanced trees either before or after mass extinction.

However, we do not believe these results show that digital evolution is inappropriate for macroevolution-related questions. Rather, we think they highlight several shortcomings of the Heard-Mooers model that limit considerably the scope of its application to evolving clades:

1. The simulation is size-based as opposed to time-based. Tree growth and trait evolution occur only until the tree reaches a specified size, a mass extinction event occurs, and recovery proceeds only until the tree has recovered its pre-extinction size. In real clades and population-based simulations (such as digital evolution), trait evolution and turnover of taxa can continue well after the tree has reached an equilibrium size (with or without mass extinction) with additional consequences for tree shape descriptors [6, 11, 44].
2. The definition of recovery is not one often employed by paleontologists, who usually define “recovery” through criteria completely external to clade size [48], such as morphospace occupation [49, 50], ecological breadth and niche occupancy [51], or levels of geochemical proxies for productivity [52]; digital evolution has analogous criteria [6, 53].
3. The Heard-Mooers model is “pure birth” both before and after the mass extinction event. Background extinction is an omnipresent feature in both real clades [37] and in digital evolution, where it results from limits on population size, ecology, resource availability, and user-defined limits on the age of individual organisms.
4. Heard and Mooers [45] only considered what changes occurred in the tree relative to its pre-extinction state. This is certainly relevant for real paleontological contexts, as pre- and post-extinction states are the only information available. Given that their modeled traits could conceivably continue to affect tree growth and shape dynamics, it is worth considering how evolution would have proceeded in the absence of the mass extinction event, and how tree shape would have differed as a result.

In this paper, we revisit the question posed by Heard and Mooers [45], but incorporating considerations from Yedid et al [6] that are also likely to have bearing on real-world situations. Specifically, we investigate the effect of mass extinction and recovery on model clades that have heritable rates of speciation and extinction, but where clade size is constrained by diversity-dependence, and where the evolution of traits—and associated speciation rates—continues past the point of clade-size recovery. Since digital evolution has potential drawbacks concerning manipulation of tree properties, we approach the problem more conventionally, using branching process models.

## Methods

### Tree simulations

We simulated tree growth with a birth-death process incorporating speciation, extinction, and constrained clade size using **MeSA** v.1.12 (www.agapow.net/software/mesa; [13, 27].

#### Basic tree growth

Each tree grew from a root node object containing a single continuous-valued trait with a starting value of 10.0. Evolutionary change in this trait was simulated in a “punctuated and Brownian” manner: at speciation, one daughter taxon simply inherited the parental trait value, while the other received a trait value taken from a normal distribution around the parental value (standard deviation of 0.3 for the simulations described here). The first speciation event was forced in order to ensure that trees did not die at the root node. Only terminal taxa that had not yet gone extinct could speciate.

#### Speciation, extinction, and tree size constraint

A base speciation probability of 0.1 per extant taxon per time unit was set as the default for all terminal taxa. Speciation probabilities were influenced by a taxon’s trait value such that the smaller the value, the higher the probability of speciation would be, using they the formula ax^b^ + c, where c is the base speciation probability, x is the taxon’s trait value, and a and b are constants. While such a model may not describe the evolution of many biological traits very well, we chose it for ease of implementation within MeSA and because it conforms to the assumption of linear change along a tree branch (compare [36]). In order to make the trait-based term the same order of magnitude as the base speciation probability, we used a = 5 and b = -2; for example a trait value of 10.0 would produce a speciation probability of 0.15. Trait values were bounded by a lower limit of 2.0 and an upper limit of 15.0 in order to prevent speciation from becoming too infrequent when the tree was not near its maximum size (see below). Background extinction occurred with a constant probability (again, see [36]) of 0.05 per time unit for all terminal taxa; values lower than this made speciation events and trait evolution too infrequent when combined with diversity-dependence (see below).

In order to avoid unbounded tree growth, but also allow evolution to continue, speciation probabilities were further modified in a diversity-dependent manner, as several previous studies have found evidence for diversity-dependence in speciation rates [9, 11, 54, 55], although this pattern may depend on ecological and geographic scale [56]. A logistic model was chosen for the form of diversity-dependence, as this has been employed in previous modeling approaches [5, 36, 56, 57]. Since the trees in the motivating study [6] were fairly large (≥ 1200 tips), we set a maximum size (768) for the number of extant terminal taxa in the tree at any given time. With diversity-dependence, speciation probabilities for all extant terminal taxa would decline the closer the number of such taxa came to this limit. Thus, tree size would fluctuate around a long-term equilibrium as there were times when the number of taxa lost to background extinction would exceed those generated by new speciation events.

#### Mass extinction events

Mass extinction was implemented as an instantaneous “pulse” event: at a particular point in the simulation, a user-specified fraction of terminal taxa were culled from the tree. Four treatments were employed, three of which follow Heard and Mooers [45]:

1. a control treatment, in which no mass extinction occurred and evolution simply continued uninterrupted;
2. Random extinction (“**Random**”), where taxa were culled without regard to trait value or phylogenetic position;
3. Selective-on-diversifiers (**SOD**), where those taxa with the lowest trait values, and consequently highest speciation rates, were culled preferentially, starting from the lowest-valued taxon present;
4. Selective-on-relicts (**SOR**), where those taxa with the highest trait values, and consequently lowest speciation rates, were culled preferentially, starting from the highest-valued taxon present.

Each of the mass extinction events occurred at intensities (denoted *μ*_*M*_) of 90% (0.9), 75% (0.75), and 50% (0.5) of all extant taxa in the tree. Following mass extinction, trees recovered and continued evolving according to the same rules that had been in effect prior to the extinction event.

We further define recovery from mass extinction to have two distinct phases: **clade-size recovery, (CSR)**, covering the time where the tree either recovers to its pre-extinction size or settles on a new equilibrium value, and **post-CSR**, the time after CSR to the end of the simulation. Clade-size recovery is not defined for the control treatment as there is no mass extinction from which to recover.

#### Time course

Simulation time was measured in a series of arbitrarily-valued “ticks” (probabilities for speciation, extinction, etc. are per tick). During each tick, the rules for trait evolution, speciation, and extinction were applied to the terminal taxa of the tree. Rule parameter values could be changed at any given time, although in practice nearly every tick had the same rules applied with the same parameter values. The only ticks for which rules differed were the first, where the initial speciation was forced (see above), and the one in which the mass extinction treatment was applied (t = 300). Every five ticks, the state of the tree (containing the complete tree structure (both extinct and extant taxa) and trait values) was saved in a NEXUS-format file. Tree states were saved both immediately before and after the mass extinction event.

For each combination of mass extinction type (4 types including control), and mass extinction strength (3 levels), we ran a series of 100 replicates, for a total of 1200 runs. By default, mass extinction events were set to occur at 300 ticks of the simulation, regardless of the state of the tree. The total length of each simulation was 600 ticks.

### Tree analysis

The NEXUS files produced by MeSA were manipulated and analyzed using functions in the **APE** [58], **phytools** [59], and **apTreeshape** [23] packages in R. For each file, the entire tree structure containing both extinct and extant taxa was extracted, and all extinct taxa removed, leaving only extant taxa for a given time point [43, 44, 60]. For each tree in a time series, balance was then determined using the *colless* function from **apTreeshape**, which calculates the well-known Colless index of imbalance ([15], here abbreviated *I*_*C*_) using the normalization of Blum et al [21]. With this normalized metric, a Yule tree has an average score of zero; trees more balanced than Yule have negative values, while those less balanced than Yule have positive values.

#### Determination of Yule tree balance limits

For assessing the degree of (im)balance of a particular tree from our simulations, the limits for balance in Yule trees of sizes similar to those we generated were assessed using functions from **apTreeshape**. The *rtreeshape* function was used to generate 25,000 random Yule trees with 400, 500, 600, 700, and 800 tips (the maximum size that could be attained in the simulations was 768), and a distribution of balance scores for each tree was determined with the *colless* function. For each of these distributions, we determined the upper and lower quartiles, as well as the lower 2.5% and upper 97.5% tails. We refer to the range of values bounded by the latter pair of values as the ***outer Yule zone***; trees with a balance score that fall within this zone are not significantly different from trees of that size that can be generated by a Yule process. The upper and lower quartiles define the boundaries of the ***inner Yule zone;*** trees with balance scores within these inner bounds are around the average expectation for a Yule process. We make this distinction because we show examples of both full trajectories of single trees, and samples of trees at particular times of interest. While these boundaries make clear the degree of (im)balance of a single tree, a sample of trees may contain a substantial number of non-Yule trees and yet be not significantly different from Yule overall (i.e. the sample’s error bars overlap the boundaries).

### Data partitioning and analysis

We first examined the average change in tree balance (as measured by *I*_*C*_) at pre-extinction, CSR, and end-simulation time points, as well as the one-quarter, midpoint, and three-quarter points between CSR and end-simulation (hereafter called the CSR/end-simulation interval times). The pre-extinction and end-simulation points were fixed with respect to time (at t = 300 and t = 600 respectively), while the other time points varied considerably among replicates and among treatments. For this reason and for data visualization, the times at which these events occurred were treated as categories rather than as a continuous variable. For example, “CSR” denotes when a recovering tree either regained its pre-extinction size or converged on a new equilibrium size, regardless of the actual time during the simulation that event actually occurred. The actual values of these times were not used in statistical analysis involving comparisons of balance, but were used for comparisons of times of the given events (see below).

#### Generation of significant imbalance

For control treatments, where mass extinction did not occur, we first noted in the data when the balance score “broke out” of the Yule zone (see S1 Data). We define a ***Yule zone breakout*** as the first of a sequence of at least five consecutive sampling times with a normalized *I*_*C*_ greater than the Yule zone’s upper boundary. We define the breakout in this way because empirically, a trajectory was unlikely to wander back within the upper Yule zone boundary once it had escaped for at least 25 ticks (five recordings of five ticks each).

#### Determination of root age

Following Yedid et al [6], we wished to determine the identity and age of the root of the tree through time, in order to see if changes in (im)balance over time were being measured according to a common reference point. As MeSA does not label internal nodes in the NEXUS-format files it produces, we could not determine root identity directly. We instead determined root age and identity indirectly using the *branching*.*times* function in **APE**. This function returns the distances between every tip and internal node, the maximum of which is the distance between the youngest extant tip and the root. The difference between this maximum value and the current time of sampling yields the age of the root. Assuming that the tree is lengthening at an approximately constant rate once it has attained an equilibrium size, we reason that as long as that difference remains approximately constant, the tree maintains the same root. A change in this difference indicates loss of the previous root through extinction of a basal clade and replacement by a younger, shallower root. Using this methodology, we recorded for each simulation the times of root replacement and the inferred age of the root at those times.

#### Among-extinction-treatment comparisons

For tree balance at CSR, the post-CSR interval times, and end-simulation times, we first analyzed the treatments involving a combination of extinction type and intensity (Random, SOD, SOR) with two-way ANOVA with Tukey-corrected multiple comparison testing, in order to see whether there were any significant treatment-by-intensity interactions. Different treatment intensities were treated as non-numerical factors, and a family-wise confidence level of 95% was assumed for all comparisons. For comparisons of key times, in order to maintain a balanced experimental design, if balance did not return to the Yule zone within the allotted time of the simulation, the time was considered the maximum length of the simulation. These analyses were performed in R v.3.2.3 (R Core Team 2015) with the *aov* and *TukeyHSD* functions.

#### Comparisons to control and pre-treatment reference points

We were also interested in whether the various extinction treatment outcomes differed systematically from the control and pre-treatment reference points. To that end, we also performed Dunnett’s tests [61], using either pre-treatment or the control treatment as the reference standard. For CSR trees, only pre-treatment was used as the reference, whereas for end-simulation, comparisons were made using both Control and pre-treatment as reference points. For comparisons involving pre-treatment as the standard, all treatments including Control were considered as single factors. These analyses were performed in R v.3.2.3 using the *glht* function (part of the **multcomp** library), using the “Dunnett” option for contrasts.

**Fig 2:**
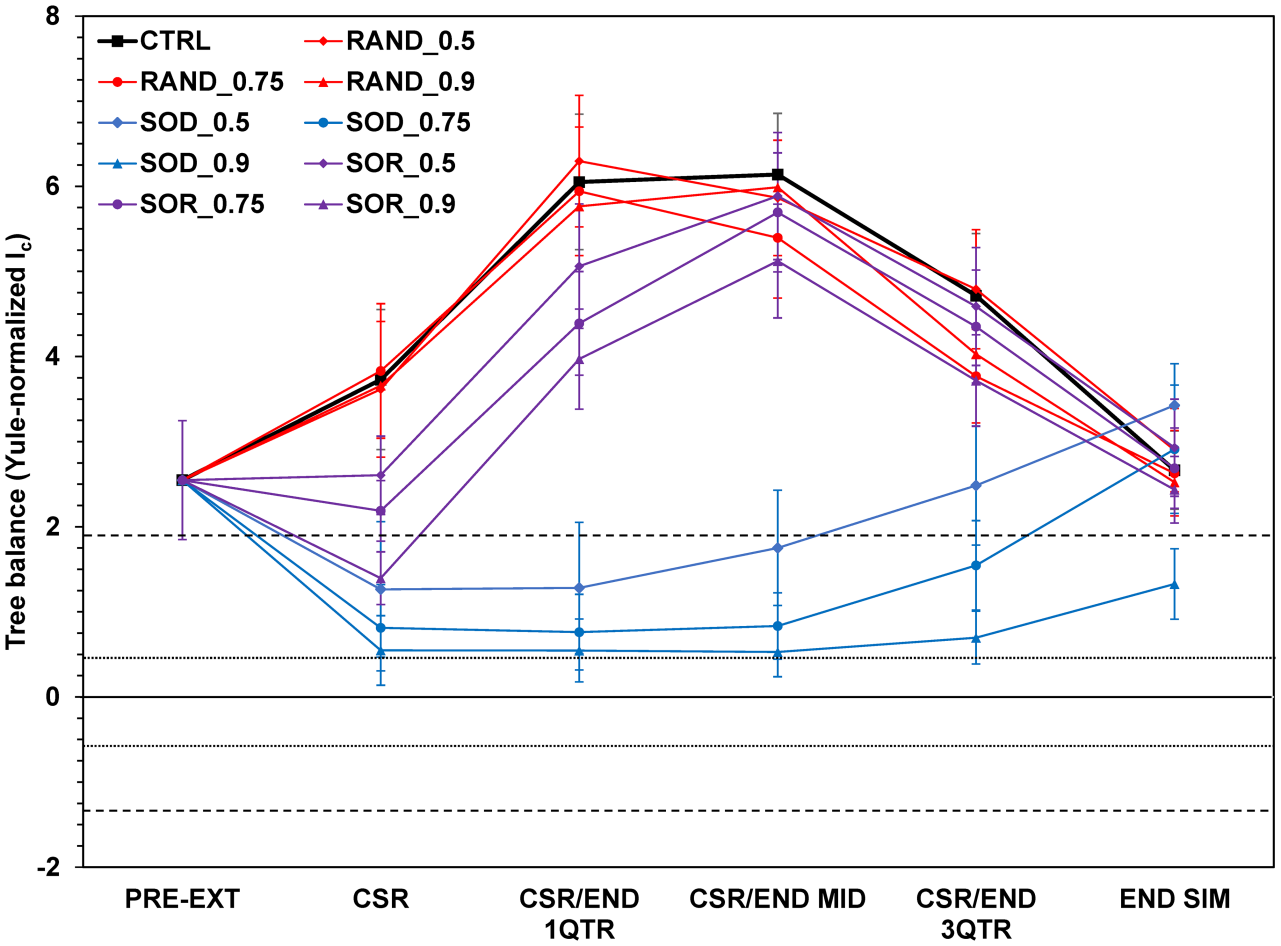
Effect of mass extinction and recovery beyond clade-size recovery using Blum et al.’s [21] Yule-standardized version of Colless’ [15] index of imbalance. CSR = clade-size recovery; CSR/END 1QTR, MID, 3QTR = CSR/end simulation first-quarter, midpoint and three-quarter points; END SIM = end-simulation. RAND = random extinction; SOD = selective-on-diversifiers; SOR = selective-on-relicts. 0.5, 0.75, and 0.9 refer to extinction intensity. Short-dashed lines above and below the zero line indicate boundaries of inner Yule zone; long-dashed lines indicate outer Yule zone boundaries. All data points are averages of 100 replicates ± 2 standard errors. See Methods for statistical analysis and special statistical treatment regarding Control.

As CSR was not defined for Control runs, we analyzed differences between treatments and control as follows for CSR and post-CSR interval times. For each treatment replicate, we determined the time of CSR, and associated value of tree balance. We then determined tree balance at the same time in the corresponding Control run. This way, each series of treatment values could be compared to a different series of Control values through conventional two-tailed t-tests. For simplicity of graphical display, we determined the approximate grand mean times for CSR and post-CSR intervals across all treatments (about t = 335, 405, 470, and 535 respectively), and found the corresponding Control values for these times; the averages of these latter values are what appear on Fig 2 as the Control points for those key times. All summary statistics are reported as means ± two standard errors.

## Results

### General trajectory of tree balance through time under trait-biased model of diversification

**Fig 3:**
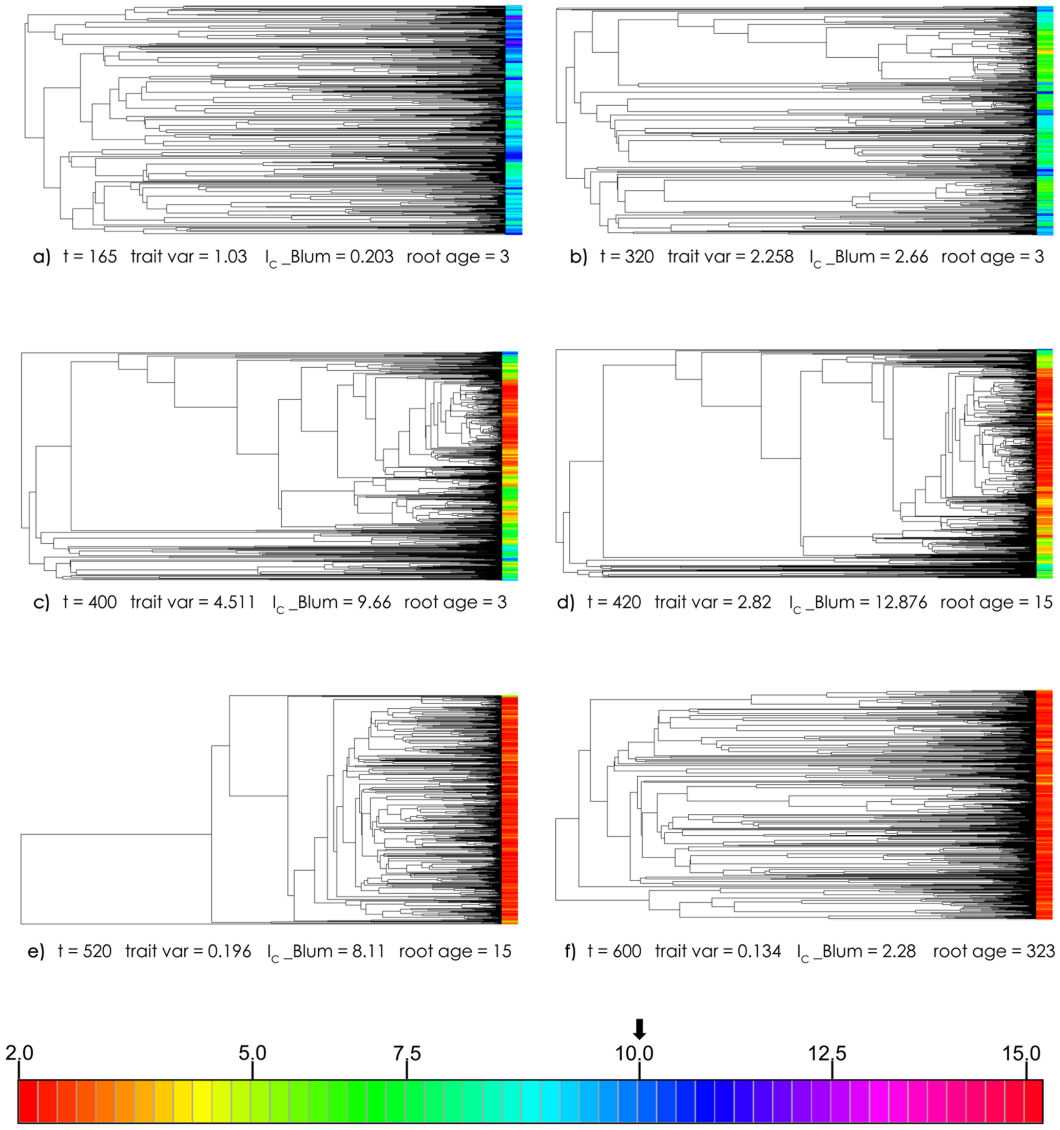
Exemplar phylogenetic trees showing change in balance and trait/rate values over time. Branch lengths are scaled in MeSA absolute time. These trees correspond to the histograms of trait variance shown in S5 Fig. Tips are coloured according to trait value ranges shown in colour scale at bottom.
a)t = 165, trait variance approximately 1, increasingb)t = 320, trait variance at half-maximum, increasingc)t = 400, maximum varianced)t = 420, half-maximum, descendinge)t = 520, variance < 1 but still strong imbalance, descending.f)t = 600, variance at end-simulation

Under the models of trait evolution and trait-biased speciation probabilities used here, evolutionary change in tree balance followed a variable, yet still stereotyped trajectory (Fig 2, S1a Fig). An exemplar Control replicate, in which the changing phylogeny is shown with changes in the trait/rate values of taxa over time, is presented in Fig 3 (the associated distributions of trait values are in S5 Fig). After an initial period of growth to equilibrium size and wandering in the Yule zone (indicating trees whose degree of balance was still insignificantly different from what a Yule process could generate), a second phase ensued whereby the degree of imbalance—measured by strongly positive values of *I*_*C*_—increased sharply over Yule zone values (the ***Yule zone breakout***).

Breakout time varied substantially among replicates (mean breakout time 319 ± 20 ticks for all replicates, 373 ± 14 for 66/100 replicates for breakout time ≥ 300 ticks), but it always occurred. Once this escape from the Yule zone occurred, tree imbalance would rise to a peak value. However, this heightened degree of imbalance was not sustainable indefinitely, as *I*_*C*_ would then decline from this peak until back within Yule zone boundaries. Even so, only 46% of replicates returned to the Yule zone within the initial allotted time of the simulation (mean time of return 527 ± 18 ticks). In S3 Data, we show in more detail how this trajectory results from the erosion of trait/rate variance when limits on trait values are reached.

When the “breakout” behaviour happened after the fixed extinction time, the extinction type and intensity could alter the time at which key events (Yule zone breakout, time of CSR, Yule zone return time) occurred. The greatest differences were associated with the SOD treatment, which substantially delayed breakout time and CSR compared to Random and SOR, but accelerated Yule zone return time (more detailed explanations and statistics given in S1 Data).

### Among-treatment differences in balance at key times in evolutionary trajectory

We focused primarily on the differences in tree balance between treatments at CSR, post-CSR interval times, and end-simulation.

Significant differences in balance among treatment/intensity combinations were found at all key times. Initial inspection of the data suggested three groups of CSR outcomes based on treatment type (Fig 2, “CSR”), with Random producing the most imbalanced trees, SOD the most balanced trees, and SOR intermediate between the other two treatment groups. Random extinction always differed significantly from SOD and from SOR at *μ*_*M*_ > = 0.75 (Table 1). Although the greatest imbalancing effect was produced by Random at *μ*_*M*_ = 0.75 (Table 2, consistent with [45]), *within* treatment types, no intensity differed significantly from any other, and model reduction indicated non-significance of the interaction term. Random did not differ significantly from corresponding Controls at any intensity, while SOR differed from Control only at the higher intensities. Only SOD differed systematically from Control (Fig 2, Table 2). We then compared balance after the mass extinction treatments to the pre-extinction state (the comparison standard); only SOD differed significantly at all intensities, Random at the two higher intensities, and SOR only at the highest intensity (Table 3).

**Table 1:** Mean differences in balance between mass extinction treatments at point of clade-size recovery.

		**RANDOM**			**SOD**			**SOR**		
		**0.5**	**0.75**	**0.9**	**0.5**	**0.75**	**0.9**	**0.5**	**0.75**	**0.9**
**RANDOM**	**0.5**	-----	0.317	0.08	2.35****	-2.66****	-2.95****	1.01	-1.42*	-2.22****
	**0.75**		-----	-0.24	2.67****	2.98****	-3.27****	1.33*	1.74***	-2.54****
	**0.9**			-----	2.43****	2.74****	3.03****	1.093	1.5**	2.23****
**SOD**	**0.5**				-----	-0.312	-0.60	-1.34*	0.93	0.13
	**0.75**					-----	-0.29	-1.65**	-1.24	0.44
	**0.9**						-----	-1.94***	-1.53**	-0.73
**SOR**	**0.5**							-----	-0.407	-1.21
	**0.75**								-----	-0.8
	**0.9**									-----

See Methods for statistical analysis. Significance levels:

no asterisk, difference not significant;

‘*’, 0.01 ≤ p < 0.05;

‘**’ 0.005 ≤ p < 0.01;

‘***’ 0.0001 ≤ p < 0.005;

‘****’ p < 0.0001.

**Table 2:** Comparison of treatments vs. corresponding control at CSR.

TREATMENT	INTENSITY	AVERAGE AT CSR	AVERAGE FOR CORRESPONDING CONTROL
Random	0.5	3.62	3.24
	0.75	3.93	3.34
	0.9	3.7	3.44
SOR	0.5	2.60	2.97
	0.75	2.19	2.96*
	0.9	1.4	3.01****
SOD	0.5	1.26	3.75****
	0.75	0.95	3.8****
	0.9	0.66	4.44****

See Methods for statistical analysis. Significance levels:

no asterisk, difference not significant;

‘*’, 0.01 ≤ p < 0.05;

‘**’ 0.005 ≤ p < 0.01;

‘***’ 0.0001 ≤ p < 0.005;

‘****’ p < 0.0001.

**Table 3:** Dunnett contrasts between tree balance at CSR for each extinction treatment and immediate pre-treatment as a reference standard.

TREATMENT	INTENSITY	Mean difference from pre-treatment (std. err. = 0.423)
Random	0.5	1.07
	0.75	1.38**
	0.9	1.15*
SOD	0.5	-1.28*
	0.75	-1.6**
	0.9	-1.89***
SOR	0.5	0.055
	0.75	-0.35
	0.9	-1.15*

See Methods for statistical analysis. Significance levels:

no asterisk, difference not significant;

‘*’, 0.01 ≤ p < 0.05;

‘**’ 0.005 ≤ p < 0.01;

‘***’ 0.0001 ≤ p < 0.005;

‘****’ p < 0.0001.

Between treatment differences decayed progressively over the course of the post-CSR period. Despite the more mildly balancing effect of SOR, imbalance continued to increase for both Random and SOR trees at all intensities up to the CSR/end first-quarter point, though SOR at *μ*_*M*_ = 0.9 still remained significantly less imbalanced than Random (Fig 2, Table D in S2 Data) and corresponding Control (Fig 2; Table E in S2 Data). SOD trees remained much more balanced, further magnifying the difference between SOD and other treatments (Fig 2; Table D in S2 Data). By CSR/end midpoint, Random and SOR no longer differed significantly from each other at any intensity (Fig 2, Table 4), and this persisted to the end of the simulation (Fig 2; Table G in S2 Data). All SOD trees remained more balanced than the other treatments, with even SOD_0.5 still distinguishable from the other treatments (Fig 2, time “CSR/END MID”; Table 4), though even this difference began to fade by the CSR/end three quarter point (Fig 2; Table G in S2 Data).

**Table 4:** Mean differences in balance between mass extinction treatments at CSR/END midpoint.

		**RANDOM**			**SOD**			**SOR**		
		**0.5**	**0.75**	**0.9**	**0.5**	**0.75**	**0.9**	**0.5**	**0.75**	**0.9**
**RANDOM**	**0.5**	----	-0.47	0.125	-4.11****	-5.03****	-5.33****	0.022	-0.17	-0.74
	**0.75**		-----	0.56	3.64****	-4.56****	-4.87****	-0.49	0.3	-0.272
	**0.9**			-----	4.237****	5.155****	-5.46****	0.103	0.296	-0.867
**SOD**	**0.5**				-----	-0.92	-1.22	4.13****	3.94****	3.37****
	**0.75**					-----	-0.305	-5.1****	4.86****	4.289****
	**0.9**						-----	-5.4****	-5.2****	4.59****
**SOR**	**0.5**							-----	-0.192	-0.764
	**0.75**								-----	-0.571
	**0.9**									-----

See Methods for statistical analysis. Significance levels:

no asterisk, difference not significant;

‘*’, 0.01 ≤ p < 0.05;

‘**’ 0.005 ≤ p < 0.01;

‘***’ 0.0001 ≤ p < 0.005;

‘****’ p < 0.0001.

For special consideration of the Control relative to treatments at CSR and post-CSR intervals (see Methods), Random differed significantly from corresponding Controls only at a few points, and then only weakly (Tables 2 and 5; Table E and Table G in S2 Data, “Random” entries). CSR values for SOR differed from corresponding Controls only at *μ*_*M*_ ≥ 0.75, while SOD differed significantly at all intensities. At post-CSR interval points, both Random and SOR tended to be less imbalanced than corresponding Controls, although any statistically significant differences were weak. Only SOD differed strongly and consistently from corresponding Control values at CSR and later points (Fig 2, Tables 2 and 5; Table E and Table G in S2 Data).

**Table 5:** Comparison between treatments vs. corresponding Control values at CSR/END midpoint.

TREATMENT	INTENSITY	AVERAGE AT CSR	AVERAGE FOR CORRESPONDING CONTROL
Random	0.5	5.86	6.50
	0.75	5.39	6.27*
	0.9	5.32	6.37*
SOR	0.5	5.63	6.42
	0.75	5.57	6.39
	0.9	5.05	6.42*
SOD	0.5	1.64	6.16****
	0.75	0.81	6.33****
	0.9	0.56	5.88****

See Methods for statistical analysis. Significance levels:

no asterisk, difference not significant;

‘*’, 0.01 ≤ p < 0.05;

‘**’ 0.005 ≤ p < 0.01;

‘***’ 0.0001 ≤ p < 0.005;

‘****’ p < 0.0001.

By end-simulation, most between-treatment differences that had existed previously had been eroded or disappeared completely, having declined from previous higher imbalance values (Fig 2, Table 6). The only significant differences all involved SOD at *μ*_*M*_ = 0.9, which remained substantially more balanced than all other treatment/intensity combinations. Most of the other treatments had, on average, converged on similar values of *I*_*C*_. The most imbalanced trees were now on average those of SOD at *μ*_*M*_ = 0.5, although the differences from other treatments were not significant. When comparing against end-simulation Control values, only SOD at *μ*_*M*_ = 0.9 differed significantly from Control (Table 7).

**Table 6:** Mean differences in balance between mass extinction treatments at end of simulation.

		**RANDOM**			**SOD**			**SOR**		
		**0.5**	**0.75**	**0.9**	**0.5**	**0.75**	**0.9**	**0.5**	**0.75**	**0.9**
**RANDOM**	**0.5**	-----	-0.27	-0.38	-0.527	0.01	-1.573***	-0.028	-0.212	-0.464
	**0.75**		-----	-0.11	-0.798	-0.282	-1.301*	-0.3	-0.059	-0.193
	**0.9**			-----	-0.904	-0.388	1.195*	-0.406	-0.165	0.087
**SOD**	**0.5**				-----	-0.516	-2.1****	0.498	-0.739	-0.991
	**0.75**					-----	-1.583***	-0.018	0.223	-0.475
	**0.9**						-----	-1.6**	-1.36*	-1.108
**SOR**	**0.5**							-----	-0.241	-0.493
	**0.75**								-----	-0.252
	**0.9**									-----

See Methods for statistical analysis. Significance levels:

no asterisk, difference not significant;

‘*’, 0.01 ≤ p < 0.05;

‘**’ 0.005 ≤ p < 0.01;

‘***’ 0.0001 ≤ p < 0.005;

‘****’ p < 0.0001.

**Table 7:** Dunnett contrasts between tree balance at end-simulation for each extinction treatment, using end-control as a reference standard.

TREATMENT	INTENSITY	End-simulation (std. err = 0.378)
Random	0.5	0.234
	0.75	-0.0374
	0.9	-0.143
SOR	0.5	0.263
	0.75	0.0217
	0.9	-0.231
SOD	0.5	0.761
	0.75	0.244
	0.9	-1.339**

See Methods for statistical analysis. Significance levels:

no asterisk, difference not significant;

‘*’, 0.01 ≤ p < 0.05;

‘**’ 0.005 ≤ p < 0.01;

‘***’ 0.0001 ≤ p < 0.005;

‘****’ p < 0.0001.

### Change in phylogenetic root

**Fig 4:**
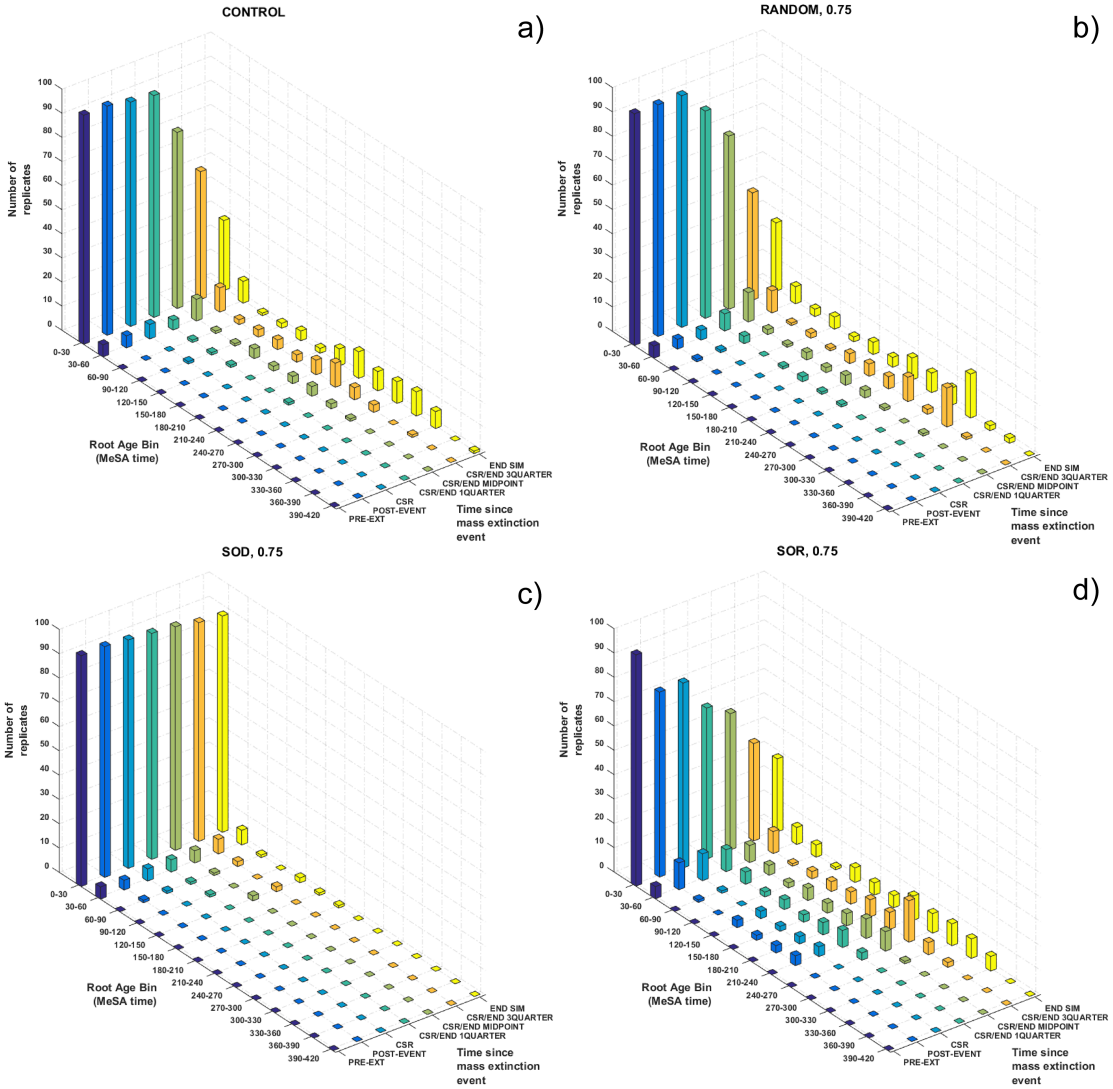
Shift in the distribution of phylogenetic root ages for a) control, b) Random at *μ*_*M*_ = 0.75, c) selective-on-diversifiers at *μ*_*M*_ = 0.75, d) selective-on-relicts at *μ*_*M*_ = 0.75. Coloured bars show number of replicates (vertical axis) with phylogenetic roots whose time of origin falls into the specified root age bins (horizontal axis). Distributions of root ages were recorded at the time points shown along the ‘‘time after extinction” axis (depth axis).

The extinction regimes differed in their effect on the age of the phylogenetic root. Just prior to treatment, 95/100 replicates had a very deep phylogenetic root, within the first 30 “ticks” of the simulation, with the remainder all originating well before the midpoint of the simulation (Fig 4a, dark blue bar series). In Control replicates, root replacement was quite common as the simulation progressed (Fig 4a). By end-simulation, the majority of replicates (85%) featured root replacements after equilibrium (mean 2.16 ± 0.312), meaning these simulations ended with a tree whose root was often substantially younger than when equilibrium size was first attained, or even the midpoint of the simulation (Fig 4a, yellow bar series). Most replacements occurred after peak trait variance and peak imbalance were attained, and were thus associated with declining imbalance and return of the tree to a Yule-like state, although not every decrease in imbalance was associated with root loss (S3a–S3c Fig).

In extinction treatment replicates, root loss depended on both treatment type and intensity. Random extinction at *μ*_*M*_ = 0.75 and 0.5 (Fig 4b, S7 Fig) generally did not replace the pre-extinction root, though mass extinction-associated root loss was more common at *μ*_*M*_ = 0.9 (S8 Fig). Further loss of deep history through background extinction produced a distribution of root ages resembling that for Control by end-simulation, though with a somewhat higher concentration of roots in the 300–330 range (Fig 4b, yellow bar series). Treatment-associated root loss was most common with SOR, which substantially reduced the number of replicates with a very deep root and spread out the distribution of root ages, particularly at high intensity (Fig 4d, compare dark blue and light blue bar series; S7c and S8c Figs). In stark contrast, SOD resulted in trees where the pre-treatment root was generally preserved over the post-extinction duration of the simulation (Fig 4c), though this effect was weakest at *μ*_*M*_ = 0.5, where the end-simulation distribution of root ages included 5 replicates with root ages ≥ 300 ticks (S7c Fig). At *μ*_*M*_ = 0.75 and *μ*_*M*_ = 0.9 respectively, 84/100 and 87/100 replicates retained the pre-extinction root by end-simulation and no replicate had a root of age ≥ 300. Thus, the tendency towards more balanced trees seen in SOD was connected with longer retention of deep history.

## Discussion

In this study, we used branching process models as implemented in MeSA to examine effects of three types of mass extinction (Random, SOD, and SOR) and recovery on tree balance, when speciation probabilities are trait-biased, heritable, and evolve in a random walk, tree size is constrained by background extinction and diversity-dependence, and recovery after mass extinction continues past the point of clade-size recovery. We showed that:

1. Tree balance followed a trajectory of initially wandering within a “Yule zone”, followed by a period of heightened imbalance strongly different from a Yule expectation, and eventual relaxation to Yule-like values.
2. Consistent with previous results, Random mass extinction generally produced more imbalanced trees after extinction and clade-size recovery (CSR) than did extinction that was selective on trait values, but did not differ significantly from corresponding Control trees. SOD extinction had a stronger tendency to result in more obviously balanced CSR trees that were generally did not differ from a Yule expectation. For SOR, stronger intensities resulted in more obviously balanced CSR trees, albeit with a weaker effect than SOD. These effects were due mostly to extinction type, rather than type-by-intensity interaction.
3. Allowing evolution to continue past the point of CSR eroded the effects of the CSR process, as Random and SOR treatments tended towards degrees of imbalance greater than that produced by mass extinction/CSR, and so would SOD after a longer time.
4. By the end of the simulation, even the additional effect of heightened imbalance had been eroded away, with most replicates converging on similar values of tree balance regardless of treatment type or intensity. Only extinction that was intensively targeted at taxa with high speciation probabilities differed from all other treatments, with most replicates remaining within the “Yule zone”.
5. Return from heightened imbalance to Yule-like values was associated with loss of deep history, often with several replacements of the phylogenetic root before the end of the simulation. Control and Random treatments behaved similarly; SOR showed the greatest amount of treatment-associated root replacement, while SOD contrasted strongly with the other treatments in showing longer retention of the pre-treatment root.

### Different extinction types display qualitatively different clade-size recovery behaviours

Our clade-size recovery results largely agree with those of Heard & Mooers [45], and the underlying causes are the same. SOD extinction removes the most actively diversifying taxa, leaving survivors with lower, more homogeneous, speciation probabilities. As traits can evolve in both directions away from the starting value, this pruning may leave a set of survivors with lower average speciation probability than the seed taxon’s. This was also shown by the notably longer CSR time for trees subjected to SOD (Table B in S1 Data). SOR leaves survivors with higher average speciation probabilities and correspondingly more rapid CSR (Table B in S1 Data). As SOR targets taxa in already-depauperate clades, post-extinction and CSR trees are relatively more imbalanced than for SOD, diluting the effect of trait/rate variance reduction. This tendency is only exacerbated upon (temporary) relief of diversity-dependent constraints, since remaining slowly-diversifying taxa are left behind by more rapid diversifiers—an effect that also occurs with Random extinction. This can be seen by the lower average CSR time of SOR compared to other treatments (Table B in S1 Data), despite the overall tendency being towards greater balance at increasing intensity (Fig 2). In contrast to trait-selective extinction, random mass extinction removes taxa irrespective of trait value or clade membership. Since Random tends to preserve more of the distribution of trait variance, the resulting imbalance is more pronounced than for SOR. When compared to Control, Random mass extinction only slightly exacerbated a tendency towards increased imbalance (Fig 2, Table 3), that was actually reversed by the selective extinction treatments (Table 3).

### Selective-on-diversifiers mass extinction leaves the most enduring signature

Our interest here goes beyond recapitulating previous results, as we wished to determine whether there is an *enduring* signature of extinction in tree balance when evolution continues past the point of CSR, similar to previous observations using one metric for tree stemminess [6]. Our results show that only SOD at high intensity consistently and reliably produced such a signature. As stated above, removal of highly-diversifying taxa may often leave speciation probabilities lower than the original seed taxon’s. Slowly-diversifying survivors must then first evolve past these reduced speciation probabilities, and regenerate sufficient trait variance in order to proceed along the breakout/return trajectory, leading to the prolonged post-extinction meandering within the Yule zone and greatly delayed breakouts we observed (Table A in S1 Data). SOD had the shortest average Yule zone return time (Table C in S1 Data) because the return is in many cases associated directly with the mass extinction itself. By contrast, Random and SOR extinctions may either advance or retard progress along the evolutionary trajectory. The overall shorter return times (Table C in S1 Data) and lower heightened imbalance (Fig 2, Table 4; Table E and Table G in S2 Data) for SOR (as compared to Control and Random) indicate that particularly at high intensity this treatment advances the trajectory past the imbalance that would otherwise be attained, moving it more quickly to a phase of greater homogeneity of trait values and speciation probabilities (albeit higher than previously), accelerating decline of imbalance and return to Yule-like values. For SOD, on the other hand, imbalance was actually increasing by the end of the simulation after remaining depressed for much of the post-CSR period, meaning many replicates had not yet reached their “true” peak imbalance.

### Continued evolution of traits and rates eventually erases the effects of different extinction types

The imbalancing effect of Random mass extinction we obtained was not enduring and not very strong considering the corresponding Control behaviour. Neither was the weaker balancing effect of SOR, and even the strong result for SOD would eventually show the same kinds of evolutionary dynamics. Indeed, the effects of mass extinction and CSR were rather weak compared to the heightened imbalance resulting from the breakout/decline behaviour. In our model, the rules of trait and rate evolution continue unchanged following mass extinction, which also temporarily relieves diversity-dependent constraints. Thus, the same phenomena that occur in Control replicates (addressed in detail in S3 Data) will eventually occur in those subjected to a mass extinction treatment; Random mass extinction disrupts those phenomena the least. The particulars will of course differ, but the long-term qualitative result will eventually be the same as Control (also see Fig 4). The factors leading to heightened imbalance and decline from it are then not consequences of mass extinction/CSR, but instead contribute to erasing the effects of the former, especially for selective extinction. Absent changes to key rules and parameters, the inevitable outcome of these processes is a return to a tree with increasingly homogenous trait/rate variance, finally erasing mass extinction/CSR-produced differences between different treatment types (Fig 2; contrast Tables 1 & 2 vs. Tables 6 & 7). Our results show that for Random, SOR, and SOD (at intermediate extinction intensity) the latter effect happens even before all replicates have completely returned to the Yule zone. Despite similar *I*_*C*_ values, the composition of taxa in the end-simulation trees differs substantially between SOD and the other treatments,

### Considering history reduces comparability of topology-based results

Although our major consideration here is of tree balance, we also considered the effects on evolutionary history using root age as a proxy [6]. Our results show that the different treatments had different short-term effects on the distribution of root ages, and that this distribution changes considerably over the course of a simulation even without mass extinction (Fig 4, S7 and S8 Figs). In many cases, tree balance is increasingly measured from different temporal reference points as a simulation progresses for Control, Random, and especially for SOR at *μ*_*M*_ ≥ 0.75. Random mass extinction had the smallest effect on root loss even at *μ*_*M*_ = 0.9, consistent with previous findings [62], but, just as with tree balance, post-extinction effects were not enduring. By contrast, SOD had a longer-lasting root-preserving effect, though even this would eventually erode once highly-diversifying taxa were regenerated. If heightened imbalance is due primarily to the retention of basal clades, and the reference point for measuring balance changes with the loss of those clades (Fig 3), the resulting tree will be phylogenetically younger and shorter in root-tip length. In our simulations, SOD generally resulted in more balanced trees with older roots, and longer retention of a common reference point for measuring balance over time. SOR tended more weakly towards more balanced trees, but these were often measured from the standpoint of a different, younger root (Fig 4, S7 and S8 Figs). Considering history and balance together, we question whether post-extinction trees are comparable to pre-treatment trees if they have lost the original reference point for balance and only more derived taxa remain [2, 50]. In such cases, then perhaps the question of the effect of mass extinction on tree balance is moot.

### Mass extinctions can contribute to, but not maintain, increased imbalance

Our results extend those of Heard and Mooers [45], examining the long-term consequences of recovery from random vs. selective mass extinctions when speciation probability is determined by a heritable trait. Our extension goes beyond re-growth of the tree to its previous size, using background extinction and diversity-dependence to produce turnover of taxa without unbounded increase in tree size and allow evolution to continue past clade-size recovery. With these additional considerations, we believe the role of mass extinction in shaping patterns of tree balance should be re-evaluated.

Although our simulations differ in some details from Heard and Mooers [45], we largely recapitulated their principal finding that random mass extinction increases tree imbalance. However, going beyond clade-size recovery shows that this is not an enduring effect *if*:

1. how traits and rates evolve remain unchanged after the mass extinction, which may be affected by altered conditions in the post-extinction world; compare the more rapid recovery from the Cretaceous-Paleogene extinction [52, 63–68] with the much slower one for the Permo-Triassic extinction [51, 69–74] (but see [75] for an interesting exception);
2. clade dynamics show diversity-dependence [4, 9, 54, 76–78]
3. trait values (and speciation rates dependent on them) are subject to limits—constraints that may be genetic [79], functional [80, 81] or ecological [82, 83] that restrict the range of feasible phenotypes;
4. there is no mechanism for isolating slowly-diversifying clades from very rapidly-diversifying ones. It is unclear whether such mechanisms in fact exist; although there are some possible examples within Primates [24, 84] and Western Hemisphere marsupials (the “possum effect” [24]), these are uncertain due to undersampling and there is no indication of this being a widespread phenomenon.

We have shown that if these conditions prevail, all treatments will eventually converge on trees with similar, Yule-like degrees of tree balance, and that strong selective-on-diversifiers extinction delays this outcome the longest. Further, random extinction only seems to add slightly to an already-existing tendency, and post-extinction tree balance often ends up being measured from different phylogenetic reference points. Thus, we believe the extent to which random mass extinctions may contribute to building the skewed pattern of diversity characteristic of many extant phylogenies (see Discussion in [45]) needs to be reconsidered.

First, we must define what it means for a clade to be “sharply imbalanced”. We can use the boundaries of the Yule zone as a reference, as a Yule process itself can generate a wide diversity of tree shapes, and trees near its upper edge can be considered already moderately imbalanced. Indeed, it can be very difficult to show that the tree of an evolving clade departs from a Yule expectation, even for paleontological time series lasting appreciable spans of geological time [41]. If it truly is the case that a sizeable majority of extant clades both tend towards sharp imbalance *and* have been subject to at least one major randomly-acting mass extinction, then attention should shift towards identifying factors that might maintain the imbalance generated by the extinction/recovery process, rather than eroding it. Also required is consideration of how long ago the last mass extinction affecting a clade occurred. To the extent that the evolution and diversification of most real-world taxa actually resembles those of simulated branching process models, asexual digital organisms, or single-celled organisms like foraminifera (whose population dynamics are most likely to resemble the former), clades for whom the most recent mass extinction event lies far in their evolutionary past may not retain much signal of short-term post-extinction diversification, especially if there has been much turnover since that time[78]. Even if a given extant clade’s phylogeny is sharply imbalanced, our results suggest this imbalance may be due to evolutionary events unrelated to the mass extinction itself.

### Results blur the distinction between micro- and macro-scales of evolution, and focus consideration on factors preserving imbalance

A further consideration is to what scale of time and biology our results best apply. Although we address an issue normally considered the domain of macroevolution, this study was inspired by a computational simulacrum of microbial experimental evolution. Indeed, many of our observations and results are understood more readily in experimental evolutionary terms, particularly as the topologies of our modeled trees are in constant flux as taxa are added and removed, and nonrandom imbalance need not be the product of singular events [44]. Speciation probability is a kind of reproductive rate, i.e. fitness, and differences between taxa in reproductive rates are analogous to differences in fitness among different clones in a population (to be clear, we are ***not*** advancing a species selection argument here). The inevitable “takeover” of the tree by taxa with higher speciation probabilities then becomes analogous to a selective sweep resulting in a shift to a population with a higher average fitness; the real-world equivalents are the suddenly greater availability of resources and space for expansion due to reduced competition following a population bottleneck, leading to a temporary burst of diversification that declines once carrying capacity is reached. The evolutionary trajectory thus reflects the success of one or a few high-fitness subclones within the “population” of taxa, initially causing sharp imbalance during the initial period of expansion, which then declines along with variance in fitness when these subclones have risen to dominance. Nor is this because of a single global fitness optimum; the two-phase simulations described in S3 Data demonstrate that this behaviour still occurs (albeit to lesser degrees) when trait limits, now analogous to new fitness optima, are accessed serially with a waiting time between them, akin to classical periodic selection [85]. For these reasons, our results apply best to phylogenies at lower levels of taxonomic resolution. We feel justified in drawing such analogies, since there is no *a priori* reason why diversifying clades of clonal asexual organisms cannot show phylogenetic dynamics like those of obligately sexual organisms as long as strictly branching (as opposed to reticulate) dynamics apply for both at some level of biological resolution. As mentioned above, our results should focus attention on what additional factors not covered in either of the models we consider can both contribute to and preserve phylogenetic imbalance. If it be the case that ecological and/or spatial isolation are needed to separate taxa with markedly different diversification rates, there is again no reason why this cannot apply to both a single initial species experiencing adaptive radiation into different spatially isolated environments, and to higher-level taxa diversifying across larger spatial scales.

### Concluding remarks

Our results again demonstrate that mass extinction that acts randomly on clades with trait-biased speciation probability produces greater imbalance than that acting in a directionally-selective manner. However, they also show that these effects are comparatively weak and short-lived when the evolutionary processes that first produce strong imbalance even without mass extinction then erodes those effects. As discussed previously [5, 6], for most real cases, we do not know where along its evolutionary trajectory a clade lies (or even what the form of the trajectory is), or what alternative outcomes could be. Other processes, such as those operating in the Control runs here, can produce sharp imbalance even without mass extinction (see also [10, 37]). Thus, we cannot solely use tree balance metrics to infer past history of mass extinction for a given extant clade’s phylogeny. Here, we know the timing and nature of the extinction events, the pre-extinction and subsequent tree states, and the behaviour that would obtain without mass extinction, including the form of the evolutionary trajectories of trait variance and tree balance. To be sure, we are not claiming that a trajectory such as the type obtained here actually characterizes most real clades, which would depend on factors not modeled here. However, our results are a further demonstration that as an evolving clade gets further away from a mass extinction event, subsequent evolution can obscure and eventually erase the initial phylogenetic effects caused by the extinction/recovery process, though this may depend on which characteristics of the phylogeny are measured, and by what metrics [6].

## Supporting information

S1 Fig Three representative replicates showing early (purple trace), middle (blue trace), and late (black trace) Yule zone breakout and return behaviour. The late-breaking replicate run was extended in order for return to the Yule zone to be clearly shown. Dot-dash vertical line at t = 300 indicates where mass extinction treatment would occur.
a)Using Blum et al.’s [21] Yule-standardized version Colless’ [15] index of imbalance. Yule zone boundaries as described in Methods.b)Using β_A_ [16]. Solid traces are maximum likelihood estimates of β_A_, dashed traces are 95% confidence intervals around the calculated β_A_ estimates. β_A_ values and confidence intervals determined with same R code as for Fig 1.
(TIF)

S2 Fig Effect of different mass extinction treatments on tree balance for the three representative replicates shown in S1 Fig (goes with S1 Data). Time of extinction treatment is t = 300 in all cases. Extinction strength is *μ*_*M*_ = 0.9 for all cases. Black trace, Control; red trace, Random; blue trace, selective-on-diversifiers; purple trace, selective-on-relicts.
a)Middle-breaker.b)Early-breakerc)Late breaker, unextended simulation. Note that selective-on-diversifiers extinction prevents Yule zone breakout within allotted time of simulation.
(TIF)

S3 Fig **(a-c). Three representative replicates showing connection between change in tree balance and loss of phylogenetic root during return to Yule zone.** Plots show behavior of Control replicates only. Top panel, trajectory of tree balance; bottom panel, change in root age. A larger root age value signifies a younger root.
(TIF)

S4 Fig **(a-c). Three representative replicates showing offset between maximum trait variance and peak imbalance (goes with S3 Data).** Black trace, tree balance; red trace, trait variance; dashed horizontal red line, trait variance = 1.0; dot-dash vertical black line, time of extinction treatment; dashed horizontal red line, time of maximum trait variance; dashed horizontal black line, time of peak imbalance.
(TIF)

S5 Fig Histograms showing shift in distribution of trait variance over time (goes with S3 Data). Figures correspond to replicate shown in S3c Fig.
a)t = 165, trait variance approximately 1, increasingb)t = 320, trait variance at half-maximum, increasingc)t = 400, maximum varianced)t = 420, half-maximum, descendinge)t = 520, variance < 1 but still strong imbalance, descending.f)t = 600, variance at end-simulation
(TIF)

S6 Fig Thirty replicates of two-phase experiments showing double peak in trait variance and offset between trait variance and imbalance peaks for each phase (goes with S3 Data). Each coloured trace is an individual replicate. Upper panel, trait variance; lower panel, tree balance.
(TIF)

S7 Fig Shift in the distribution of phylogenetic root ages for a) Random at *μ*_*M*_ = 0.5, b) selective-on-diversifiers at *μ*_*M*_ = 0.5, c) selective-on-relicts at *μ*_*M*_ = 0.5. Bars and axes as in main text Fig 4.
(TIF)

S8 Fig Shift in the distribution of phylogenetic root ages for a) Random at *μ*_*M*_ = 0.9, b) selective-on-diversifiers at *μ*_*M*_ = 0.9, c) selective-on-relicts at *μ*_*M*_ = 0.9. Bars and axes as in main text Fig 4.
(TIF)

S1 Data Effects of mass extinction treatments on Yule zone breakout times, Yule zone return times, and time of clade-size recovery. Contains Tables A-C.
**Table A. Average times of Yule zone breakout (see text for definition) for each treatment type (goes with S1 Data).** All quantities are in simulation time units, expressed as averages ± 2 standard errors. Number to left of pipe character is for treatment; number to right of pipe is for corresponding Control replicates. Number in square brackets indicates number of treatment replicates in which event occurred.
**Table B. Average times of three critical points for each mass extinction treatment (goes with S1 Data).** All quantities are in simulation time units, expressed as averages ± 2 standard errors.
**Table C**. **Average times of Yule zone return (see text for definition) for each treatment type (goes with S1 Data).** All quantities are in simulation time units, expressed as averages ± 2 standard errors. Number to left of pipe character is for treatment; number to right of pipe is for corresponding Control replicates. Number in square brackets indicates number of treatment replicates in which event occurred.
(DOCX)

S2 Data Comparison of tree balance in mass extinction treatments to corresponding controls at clade-size recovery and CSR/end interval points. Contains Tables D-G.
**Table D (goes with S2 Data). Mean differences in balance between mass extinction treatments at CSR/end first-quarter point.** See Methods for statistical analysis. Significance levels: no asterisk, difference not significant; ‘*’, 0.01 ≤ p < 0.05; ‘**’ 0.005 ≤ p < 0.01; ‘***’ 0.0001 ≤ p < 0.005; ‘****’ p < 0.0001.
**Table E (goes with S2 Data). Comparison of treatments vs. corresponding Control at CSR/end first-quarter point. See Methods for statistical analysis.** Significance levels: no asterisk, difference not significant; ‘*’, 0.01 ≤ p < 0.05; ‘**’ 0.005 ≤ p < 0.01; ‘***’ 0.0001 ≤ p < 0.005; ‘****’ p < 0.0001.
**Table F (goes with S2 Data). Mean differences in balance between mass extinction treatments at CSR/end three-quarter point.** See Methods for statistical analysis. Significance levels: no asterisk, difference not significant; ‘*’, 0.01 ≤ p < 0.05; ‘**’ 0.005 ≤ p < 0.01; ‘***’ 0.0001 ≤ p < 0.005; ‘****’ p < 0.0001.
**Table G (goes with S2 Data) Comparison of treatments vs. corresponding Control at CSR/end three-quarter point.** See Methods for statistical analysis. Significance levels: no asterisk, difference not significant; ‘*’, 0.01 ≤ p < 0.05; ‘**’ 0.005 ≤ p < 0.01; ‘***’ 0.0001 ≤ p < 0.005; ‘****’ p < 0.0001.
(DOCX)

S3 Data Yule zone breakout and peak imbalance behaviour are linked to trait value limits and exhaustion of trait variance. (DOCX)
