# Correction: The tree balance signature of mass extinction is erased by continued evolution in clades of constrained size with trait-dependent speciation

**DOI:** 10.1371/journal.pone.0197191

**Published:** 2018-05-07

**Authors:** Guan-Dong Yang, Paul-Michael Agapow, Gabriel Yedid

In the Funding section, the grant number from the funder National Natural Science Foundation of China is listed incorrectly. The correct grant number is: 31470435.

## References

[1] Yang G-D, Agapow P-M, Yedid G (2017) The tree balance signature of mass extinction is erased by continued evolution in clades of constrained size with trait-dependent speciation. PLoS ONE 12(6): e0179553 https://doi.org/10.1371/journal.pone.0179553 28644846 10.1371/journal.pone.0179553 PMC5482465

