# Supplementary figures and images for: The tree balance signature of mass extinction is erased by continued evolution in clades of constrained size with trait-dependent speciation
Source: PLoS One. 2017 Jun 23;12(6):e0179553. doi: 10.1371/journal.pone.0179553 (PMC5482465; doi:10.1371/journal.pone.0179553)

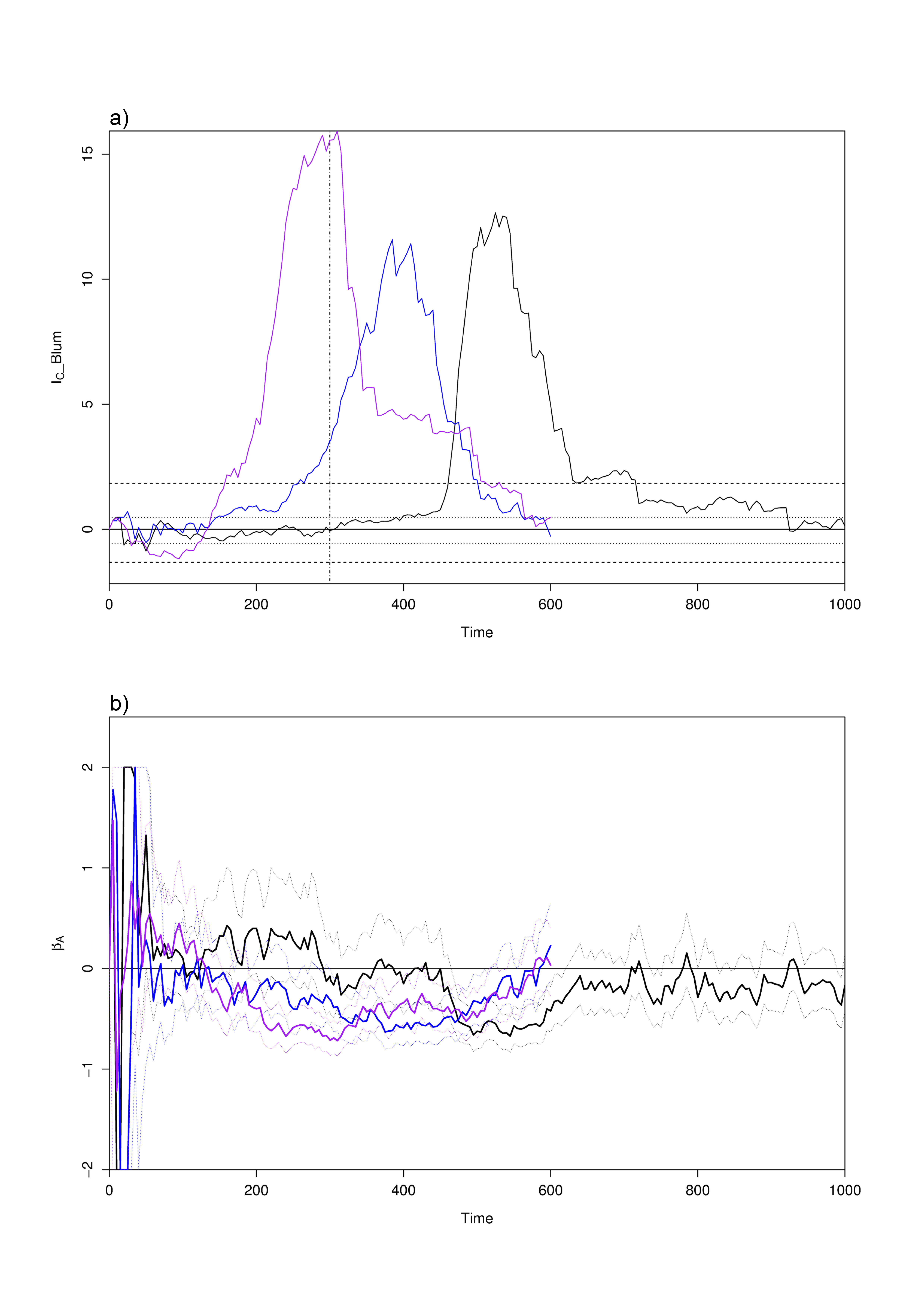

Supplement: S1 Fig — The late-breaking replicate run was extended in order for return to the Yule zone to be clearly shown. Dot-dash vertical line at t = 300 indicates where mass extinction treatment would occur. a)Using Blum et al.’s [21] Yule-standardized version Colless’ [15] index of imbalance. Yule zone boundaries as described in Methods.b)Using βA [16]. Solid traces are maximum likelihood estimates of βA, dashed traces are 95% confidence intervals around the calculated βA estimates. βA values and confidence intervals determined with same R code as for Fig 1. (TIF) [file pone.0179553.s001.tif]

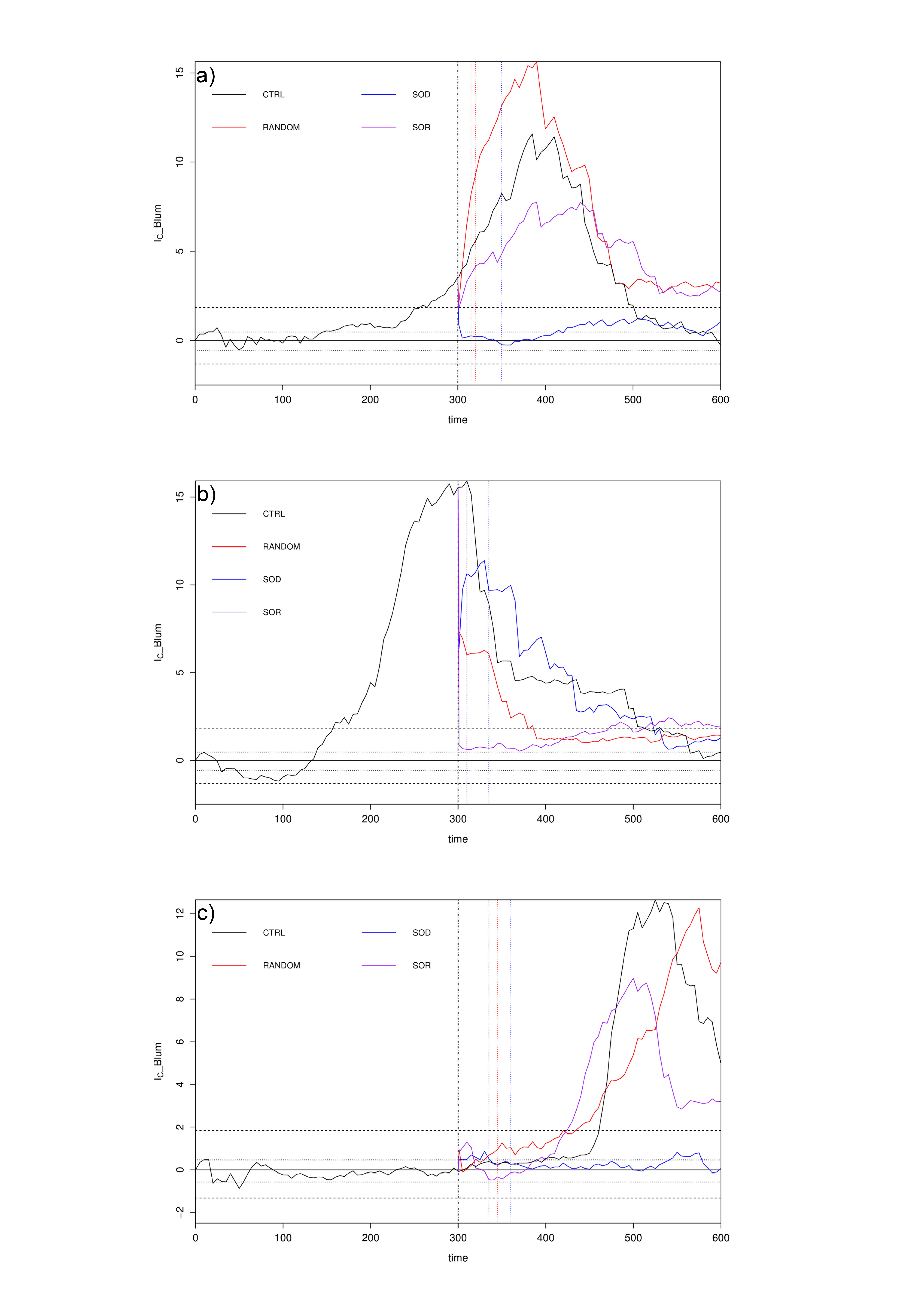

Supplement: S2 Fig — Time of extinction treatment is t = 300 in all cases. Extinction strength is μM = 0.9 for all cases. Black trace, Control; red trace, Random; blue trace, selective-on-diversifiers; purple trace, selective-on-relicts. a)Middle-breaker.b)Early-breakerc)Late breaker, unextended simulation. Note that selective-on-diversifiers extinction prevents Yule zone breakout within allotted time of simulation. (TIF) [file pone.0179553.s002.tif]

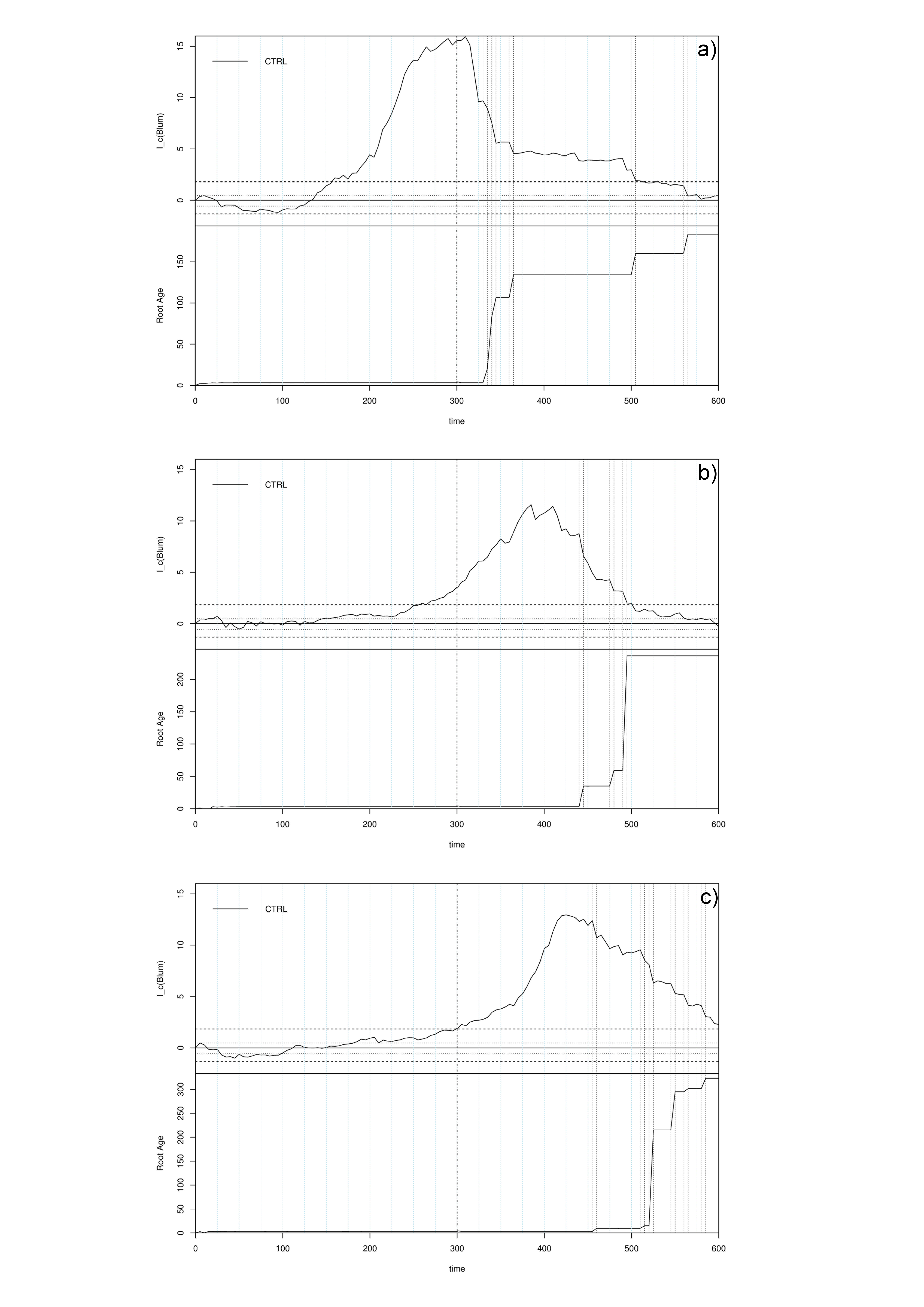

Supplement: S3 Fig — (a-c). Three representative replicates showing connection between change in tree balance and loss of phylogenetic root during return to Yule zone. Plots show behavior of Control replicates only. Top panel, trajectory of tree balance; bottom panel, change in root age. A larger root age value signifies a younger root. (TIF) [file pone.0179553.s003.tif]

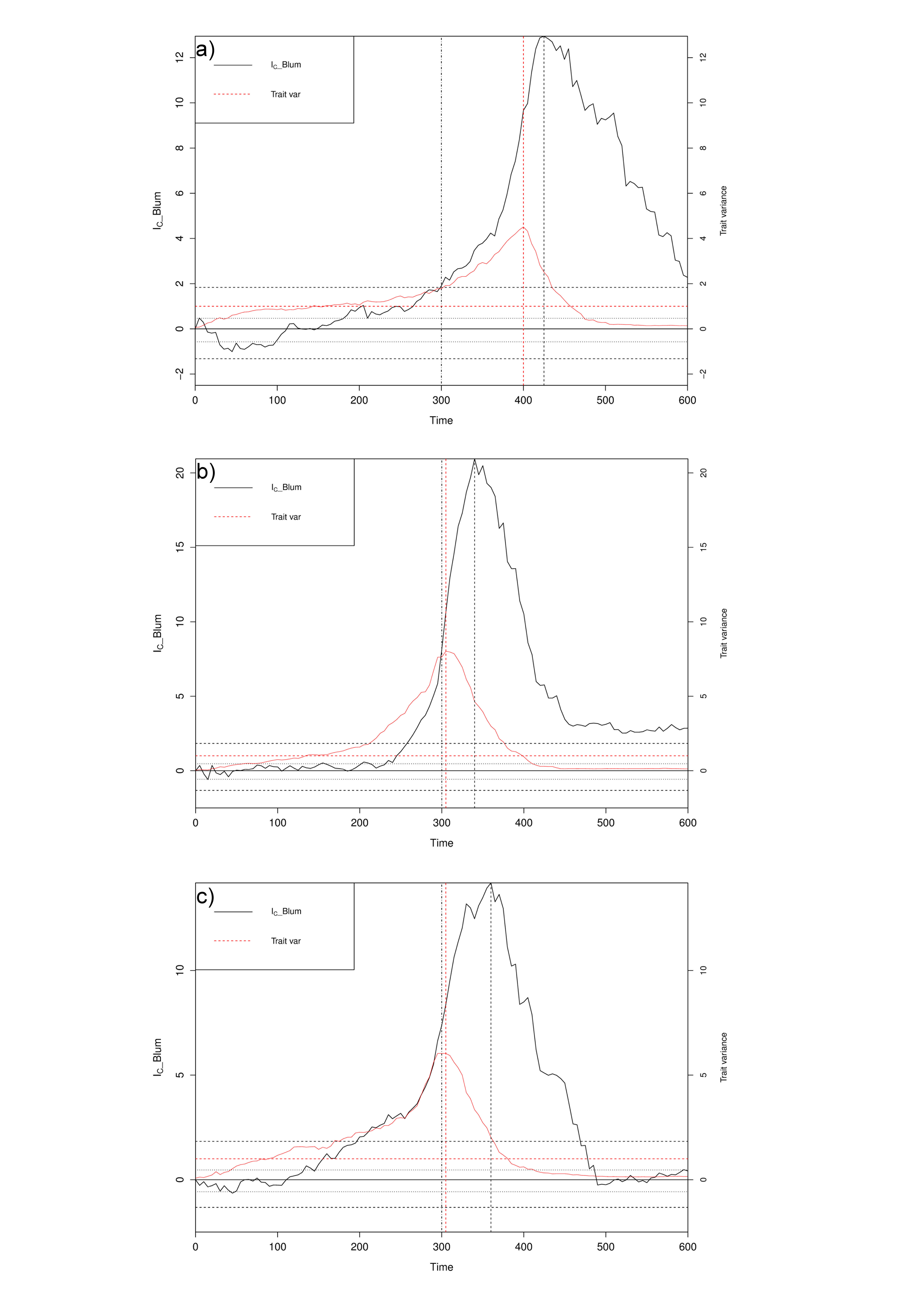

Supplement: S4 Fig — (a-c). Three representative replicates showing offset between maximum trait variance and peak imbalance (goes with S3 Data). Black trace, tree balance; red trace, trait variance; dashed horizontal red line, trait variance = 1.0; dot-dash vertical black line, time of extinction treatment; dashed horizontal red line, time of maximum trait variance; dashed horizontal black line, time of peak imbalance. (TIF) [file pone.0179553.s004.tif]

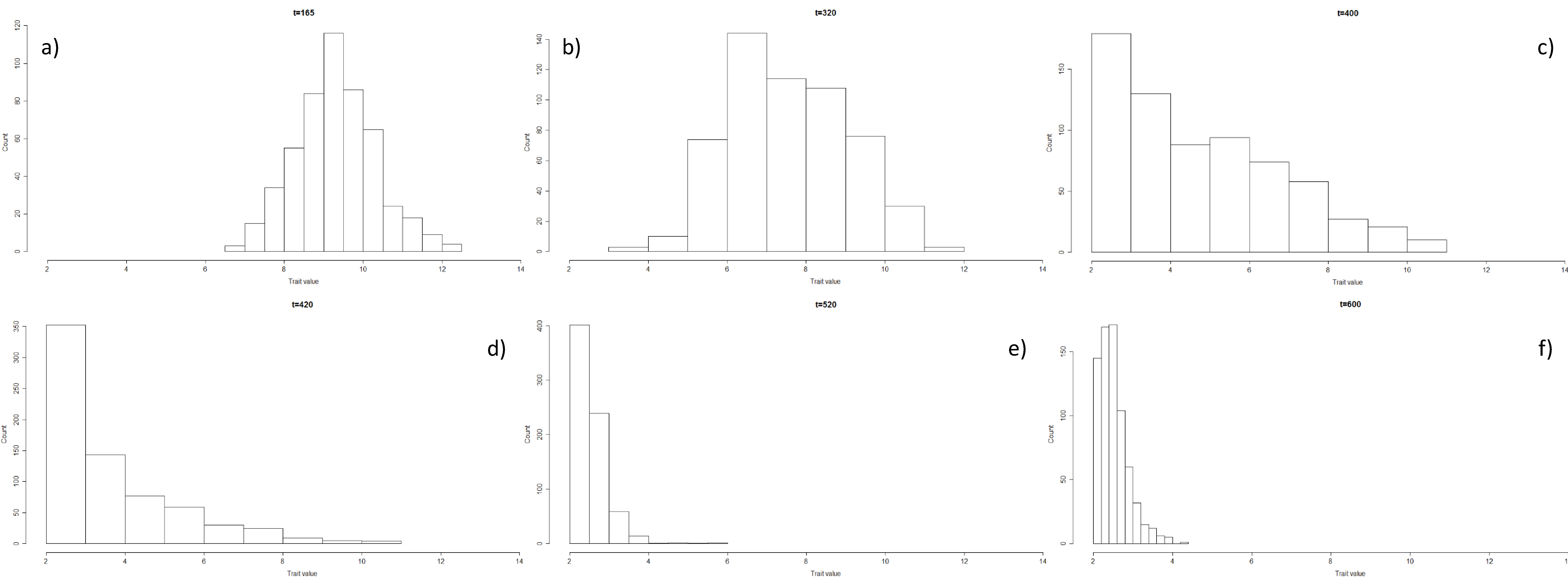

Supplement: S5 Fig — Figures correspond to replicate shown in S3c Fig. a)t = 165, trait variance approximately 1, increasingb)t = 320, trait variance at half-maximum, increasingc)t = 400, maximum varianced)t = 420, half-maximum, descendinge)t = 520, variance < 1 but still strong imbalance, descending.f)t = 600, variance at end-simulation (TIF) [file pone.0179553.s005.tif]

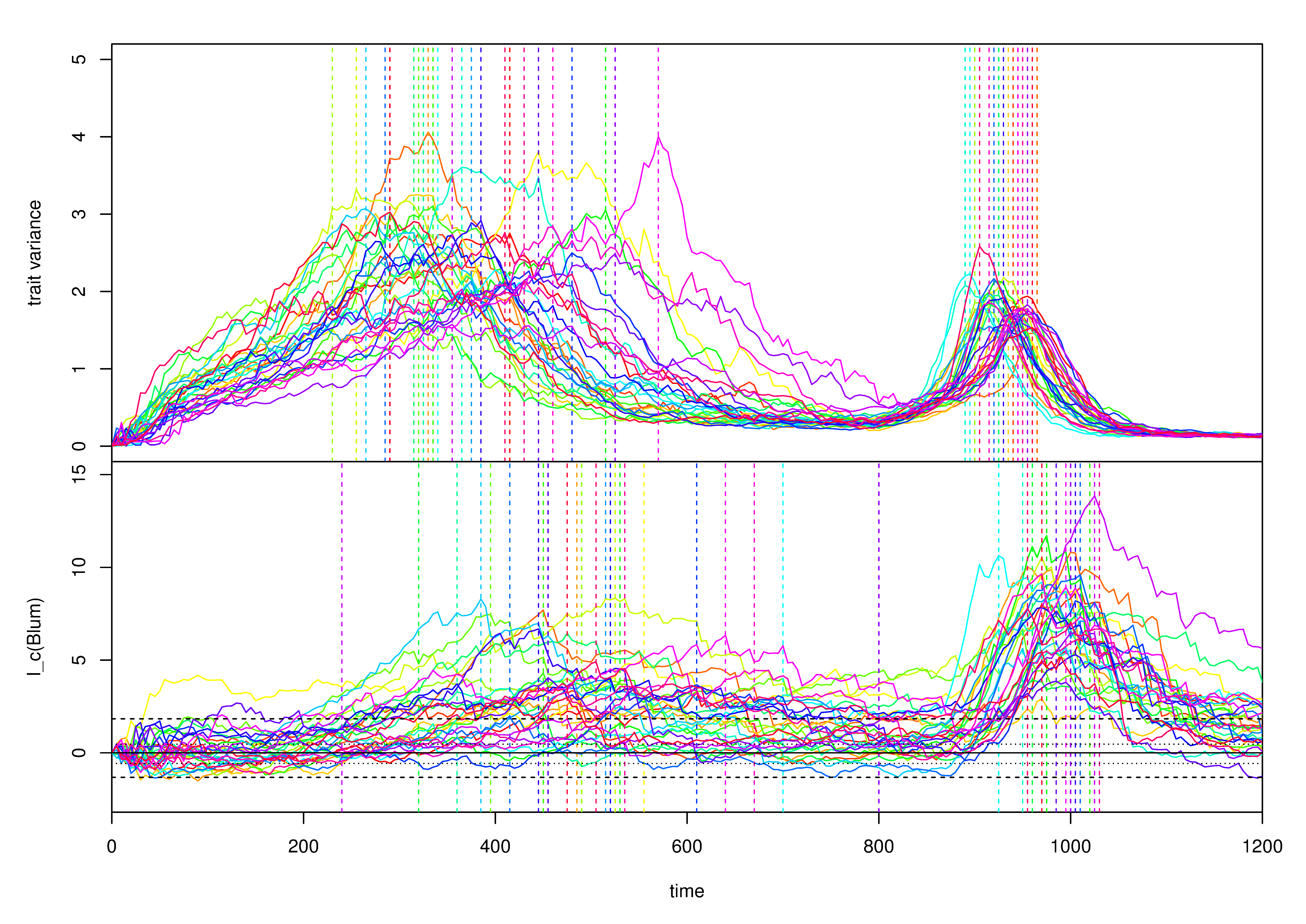

Supplement: S6 Fig — Each coloured trace is an individual replicate. Upper panel, trait variance; lower panel, tree balance. (TIF) [file pone.0179553.s006.tif]
