# Supplementary material for: The tree balance signature of mass extinction is erased by continued evolution in clades of constrained size with trait-dependent speciation
Source: PLoS One. 2017 Jun 23;12(6):e0179553. doi: 10.1371/journal.pone.0179553 (PMC5482465; doi:10.1371/journal.pone.0179553)
Supplement: S1 Data — Contains Tables A-C. Table A. Average times of Yule zone breakout (see text for definition) for each treatment type (goes with S1 Data). All quantities are in simulation time units, expressed as averages ± 2 standard errors. Number to left of pipe character is for treatment; number to right of pipe is for corresponding Control replicates. Number in square brackets indicates number of treatment replicates in which event occurred. Table B. Average times of three critical points for each mass extinction treatment (goes with S1 Data). All quantities are in simulation time units, expressed as averages ± 2 standard errors. Table C. Average times of Yule zone return (see text for definition) for each treatment type (goes with S1 Data). All quantities are in simulation time units, expressed as averages ± 2 standard errors. Number to left of pipe character is for treatment; number to right of pipe is for corresponding Control replicates. Number in square brackets indicates number of treatment replicates in which event occurred. (DOCX) [file pone.0179553.s009.docx]

SUPPLEMENTARY DATA S1—EFFECTS OF MASS EXTINCTION TREATMENTS ON YULE ZONE BREAKOUT TIMES, YULE ZONE RETURN TIMES, AND TIME OF CLADE-SIZE RECOVERY

When the “breakout” behaviour happened after the fixed extinction time, the extinction type and intensity could alter the time at which key events occurred. Random and SOR treatments altered the breakout time, although the differences were clearly not significant at any extinction intensity (Table 1). This was because there was no tendency for the breakout times in these treatments to be either systematically earlier or later than the corresponding Control breakout times, and a number of the breakouts were a direct result of the mass extinction itself. In stark contrast, SOD acted to greatly delay, or even suppress breakouts. This was particularly true at *μ_M_* = 0.9, where 57/100 replicates did not escape the Yule zone over the initial allotted time of the simulation. Of 9/100 that did break the Yule zone following the SOD mass extinction, 2 were a direct result of the extinction itself, while the other 7 happened very late in the simulation compared to corresponding controls (567.86 ± 21.25 ticks for SOD_0.9 vs. 351.11 ± 29.57 ticks for Control). At *μ_M_* = 0.75, 40/100 replicates failed to break out following the extinction treatment. Of the 26/100 replicates that did, the breakout time was again, on average, considerably delayed compared to the corresponding Control replicates (524.12 ± 29.27 for SOD_0.75 vs. 359.42 ± 26.66 ticks for Control). Effects of mass extinction on several exemplar replicates are shown in Fig S2.

**Fig S2. Effect of different mass extinction treatments on tree balance for the three representative replicates shown in Fig S1. Time of extinction treatment is t=300 in all cases. Extinction strength is µ_M_ = 0.9 for all cases. Black trace, Control; red trace, Random; blue trace, selective-on-diversifiers; purple trace, selective-on-relicts.**

1. **Middle-breaker.**
2. **Early-breaker**
3. **Late breaker, unextended simulation. Note that selective-on-diversifiers extinction prevents Yule zone breakout within allotted time of simulation.**

**Supplementary Table A. Average times of Yule zone breakout (see text for definition) for each treatment type. All quantities are in simulation time units, expressed as averages ± 2 standard errors. Number to left of pipe character is for treatment; number to right of pipe is for corresponding Control replicates. Number in square brackets indicates number of treatment replicates in which event occurred.**

|  | 0.5 | 0.75 | 0.9 |
| --- | --- | --- | --- |
| CONTROL, all runs | 319.2 ± 19.49 [100] |  |  |
| CONTROL, post extinction only | 373.33 ± 14.13 |  |  |
| RANDOM, post extinction only | 371.55 ± 14.62 \|  381.96 ± 14.94 [63] | 382.02 ± 19.03 \|  376.02 ± 14.45 [63] | 361.47 ± 14.5 \|  374.6 ± 14.67 [63] |
| SOD, post extinction only | 518.39 ± 17.74 \|  357.5 ± 19.6 [28] | 524.12 ± 29.26 \|  359.42 ± 26.66 [26] | 534.5 ± 74 \|  351.11 ± 29.57 [9] |
| SOR, post extinction only | 370.26 ± 16.87 \|  378.52 ± 14.2 [61] | 366.71 ± 17.11 \|  370.38 ± 14.7 [62] | 368.89 ± 15.12 \|  374.38 ± 14.39 [64] |

The different mass extinction treatments also affected times of CSR and Yule zone return. On average, SOR took the shortest time for CSR at all intensities, while SOD took the longest (Table 2). For those replicates that did return to the Yule zone in the allotted time, both Random and SOR substantially reduced the time needed for balance to decline to Yule-like values as well, compared to Control values (Table 3). The SOD treatment most strongly reduced time to Yule zone re-entry compared to corresponding Controls, but for a different reason: for this treatment, Yule zone re-entry was often co-incident with the mass extinction event itself, rather than an outcome of the post-CSR recovery process.

**Supplementary Table B. Average times of three critical points for each mass extinction treatment. All quantities are in simulation time units, expressed as averages ± 2 standard errors.**

| **CSR** | | | |
| --- | --- | --- | --- |
|  | **0.5** | **0.75** | **0.9** |
| RANDOM | 326.75 ± 2.71 | 329.80 ± 2.97 | 333.67 ± 3.64 |
| SOD | 342.25 ± 4.96 | 355.35 ± 6.17 | 368.2 ± 5.72 |
| SOR | 319.85 ± 1.87 | 323.91 ± 5.59 | 321.7 ± 1.85 |
|  | | | |
| **CSR/END SIM 1QUARTER POINT** | | | |
|  | **0.5** | **0.75** | **0.9** |
| RANDOM | 397.15 ±2.04 | 399.1 ± 2.22 | 401.65 ± 2.15 |
| SOD | 408.85 ± 3.73 | 416.35 ± 3.74 | 426.55 ± 3.17 |
| SOR | 391.8 ± 1.42 | 392.75 ± 1.60 | 393.25 ± 1.45 |
|  | | | |
| **CSR/END SIMULATION MIDPOINT** | | | |
|  | **0.5** | **0.75** | **0.9** |
| RANDOM | 464.45 ± 1.43 | 466 ± 1.59 | 467.6 ± 1.51 |
| SOD | 472.45 ± 2.5 | 477.55 ± 2.51 | 484.35 ± 2.1 |
| SOR | 461.2 ± 0.9 | 461.95 ± 1.03 | 462.05 ± 0.99 |
| CSR/END SIMULATION 3QUARTER POINT | | | |
|  | **0.5** | **0.75** | **0.9** |
| RANDOM | 533.65 ± 0.74 | 534.35 ± 0.79 | 535.2 ± 0.78 |
| SOD | 537.55 ± 1.26 | 540.05 ± 1.31 | 543.35 ± 1.1 |
| SOR | 532.0 ± 0.51 | 532.25 ± 0.54 | 532.35 ± 0.5 |

**Supplementary Table C. Average times of Yule zone return (see text for definition) for each treatment type. All quantities are in simulation time units, expressed as averages ± 2 standard errors. Number to left of pipe character is for treatment; number to right of pipe is for corresponding Control replicates. Number in square brackets indicates number of treatment replicates in which event occurred.**

|  | 0.5 | 0.75 | 0.9 |
| --- | --- | --- | --- |
| CONTROL, all runs | 526.63 ± 17.70 [46] |  |  |
| CONTROL, post extinction only |  |  |  |
| RANDOM, post extinction only | 531.19 ± 16.78 \|  558.69 ± 16.08 [42] | 510.93.12 ± 19.82 \|  557.11 ± 16.75 [57] | 493.27 ± 25.57 \|  555.73 ± 16.46 [63] |
| SOD, post extinction only | 383.36 ± 46.33 \|  543.6 ± 28.19 [25] | 341.45 ± 32.19 \|  540.0 ± 27.65 [31] | 315.22 ± 15.69 \|  537.34 ± 15.68 [32] |
| SOR, post extinction only | 504.9 ± 25.83 \|  549.0 ± 18.52 [50] | 458.06 ± 31.69 \|  549.06 ± 19.56 [48] | 432.49 ± 34.24 \|  555.82 ± 17.27 [55] |
