# Supplementary material for: The tree balance signature of mass extinction is erased by continued evolution in clades of constrained size with trait-dependent speciation
Source: PLoS One. 2017 Jun 23;12(6):e0179553. doi: 10.1371/journal.pone.0179553 (PMC5482465; doi:10.1371/journal.pone.0179553)
Supplement: S2 Data — Contains Tables D-G. Table D (goes with S2 Data). Mean differences in balance between mass extinction treatments at CSR/end first-quarter point. See Methods for statistical analysis. Significance levels: no asterisk, difference not significant; ‘*’, 0.01 ≤ p < 0.05; ‘**’ 0.005 ≤ p < 0.01; ‘***’ 0.0001 ≤ p < 0.005; ‘****’ p < 0.0001. Table E (goes with S2 Data). Comparison of treatments vs. corresponding Control at CSR/end first-quarter point. See Methods for statistical analysis. Significance levels: no asterisk, difference not significant; ‘*’, 0.01 ≤ p < 0.05; ‘**’ 0.005 ≤ p < 0.01; ‘***’ 0.0001 ≤ p < 0.005; ‘****’ p < 0.0001. Table F (goes with S2 Data). Mean differences in balance between mass extinction treatments at CSR/end three-quarter point. See Methods for statistical analysis. Significance levels: no asterisk, difference not significant; ‘*’, 0.01 ≤ p < 0.05; ‘**’ 0.005 ≤ p < 0.01; ‘***’ 0.0001 ≤ p < 0.005; ‘****’ p < 0.0001. Table G (goes with S2 Data) Comparison of treatments vs. corresponding Control at CSR/end three-quarter point. See Methods for statistical analysis. Significance levels: no asterisk, difference not significant; ‘*’, 0.01 ≤ p < 0.05; ‘**’ 0.005 ≤ p < 0.01; ‘***’ 0.0001 ≤ p < 0.005; ‘****’ p < 0.0001. (DOCX) [file pone.0179553.s010.docx]

SUPPLEMENTARY DATA S2—COMPARISON OF TREE BALANCE IN MASS EXTINCTION TREATMENTS TO CORRESPONDING CONTROLS AT CLADE-SIZE RECOVERY AND CSR/END INTERVAL POINTS

**Supplementary Table D. Mean differences in balance between mass extinction treatments at CSR/end first-quarter point. See Methods for statistical analysis. Significance levels: no asterisk, difference not significant; ‘*’, 0.01 ≤ p < 0.05; ‘**’ 0.005 ≤ p < 0.01; ‘***’ 0.0001 ≤ p < 0.005; ‘****’ p < 0.0001.**

|  | | **RANDOM** | | | **SOD** | | | **SOR** | | |
| --- | --- | --- | --- | --- | --- | --- | --- | --- | --- | --- |
|  |  | **0.5** | **0.75** | **0.9** | **0.5** | **0.75** | **0.9** | **0.5** | **0.75** | **0.9** |
| **RANDOM** | **0.5** | ---- | -0.35 | -0.53 | -5.02**** | -5.53**** | -5.75**** | -1.235 | -1.91*** | -2.33**** |
|  | **0.75** |  | ----- | -0.18 | 4.66**** | -5.18**** | -5.4**** | 0.88 | 1.55* | 1.97*** |
|  | **0.9** |  |  | ----- | 4.48**** | 5**** | -5.22**** | 0.704 | 1.375 | -1.79** |
| **SOD** | **0.5** |  |  |  | ----- | -0.52 | -0.73 | 3.78**** | 3.11**** | 2.69**** |
|  | **0.75** |  |  |  |  | ----- | -0.22 | -4.3**** | 3.63**** | 3.21**** |
|  | **0.9** |  |  |  |  |  | ----- | -4.5**** | -3.8**** | 3.42**** |
| **SOR** | **0.5** |  |  |  |  |  |  | ----- | -0.67 | -1.09 |
|  | **0.75** |  |  |  |  |  |  |  | ----- | -0.42 |
|  | **0.9** |  |  |  |  |  |  |  |  | ----- |

**Supplementary Table E. Comparison of treatments vs. corresponding Control at CSR/end first-quarter point. See Methods for statistical analysis. Significance levels: no asterisk, difference not significant; ‘*’, 0.01 ≤ p < 0.05; ‘**’ 0.005 ≤ p < 0.01; ‘***’ 0.0001 ≤ p < 0.005; ‘****’ p < 0.0001.**

| TREATMENT | INTENSITY | AVG AT CSR/END  1QUARTER | AVG FOR  CORRESPONDING  CONTROL |
| --- | --- | --- | --- |
| Random | 0.5 | 6.296 | 6.124 |
|  | 0.75 | 5.941 | 6.182 |
|  | 0.9 | 5.765 | 6.319 |
| SOR | 0.5 | 5.061 | 5.938 |
|  | 0.75 | 4.389 | 5.874*** |
|  | 0.9 | 3.970 | 5.882**** |
| SOD | 0.5 | 1.280 | 5.841**** |
|  | 0.75 | 0.762 | 6.264**** |
|  | 0.9 | 0.546 | 6.423**** |

**Supplementary Table F. Mean differences in balance between mass extinction treatments at CSR/end three-quarter point. See Methods for statistical analysis. Significance levels: no asterisk, difference not significant; ‘*’, 0.01 ≤ p < 0.05; ‘**’ 0.005 ≤ p < 0.01; ‘***’ 0.0001 ≤ p < 0.005; ‘****’ p < 0.0001.**

|  | | **RANDOM** | | | **SOD** | | | **SOR** | | |
| --- | --- | --- | --- | --- | --- | --- | --- | --- | --- | --- |
|  |  | **0.5** | **0.75** | **0.9** | **0.5** | **0.75** | **0.9** | **0.5** | **0.75** | **0.9** |
| **RANDOM** | **0.5** | ---- | -1.02 | -0.76 | -2.31**** | -3.24**** | -4.09**** | -0.203 | -0.44 | -1.07 |
|  | **0.75** |  | ----- | 0.26 | 1.29 | -2.22**** | -3.07**** | -0.82 | -0.58 | -0.051 |
|  | **0.9** |  |  | ----- | -1.54** | 2.48**** | -3.33**** | -0.56 | -0.32 | -0.31 |
| **SOD** | **0.5** |  |  |  | ----- | -0.937 | -1.79*** | 2.1*** | 1.87*** | 1.23 |
|  | **0.75** |  |  |  |  | ----- | -0.85 | -3.04**** | 2.81**** | 2.17*** |
|  | **0.9** |  |  |  |  |  | ----- | -3.89**** | -3.65**** | 3.02**** |
| **SOR** | **0.5** |  |  |  |  |  |  | ----- | -0.235 | -0.87 |
|  | **0.75** |  |  |  |  |  |  |  | ----- | -0.63 |
|  | **0.9** |  |  |  |  |  |  |  |  | ----- |

**Supplementary Table G. Comparison of treatments vs. corresponding Control at CSR/end three-quarter point. See Methods for statistical analysis. Significance levels: no asterisk, difference not significant; ‘*’, 0.01 ≤ p < 0.05; ‘**’ 0.005 ≤ p < 0.01; ‘***’ 0.0001 ≤ p < 0.005; ‘****’ p < 0.0001.**

| TREATMENT | INTENSITY | AVG AT CSR | AVG FOR  CORRESPONDING  CONTROL |
| --- | --- | --- | --- |
| Random | 0.5 | 5.864 | 6.503 |
|  | 0.75 | 5.394 | 6.273* |
|  | 0.9 | 5.319 | 6.372* |
| SOR | 0.5 | 5.629 | 6.419 |
|  | 0.75 | 5.571 | 6.39 |
|  | 0.9 | 5.046 | 6.418* |
| SOD | 0.5 | 1.637 | 6.163**** |
|  | 0.75 | 0.808 | 6.327**** |
|  | 0.9 | 0.558 | 5.882**** |
