# Supplementary material for: The tree balance signature of mass extinction is erased by continued evolution in clades of constrained size with trait-dependent speciation
Source: PLoS One. 2017 Jun 23;12(6):e0179553. doi: 10.1371/journal.pone.0179553 (PMC5482465; doi:10.1371/journal.pone.0179553)
Supplement: S3 Data — (DOCX) [file pone.0179553.s011.docx]

SUPPLEMENTARY DATA S3 - YULE ZONE BREAKOUT AND PEAK IMBALANCE BEHAVIOUR ARE LINKED TO TRAIT VALUE LIMITS AND EXHAUSTION OF TRAIT VARIANCE

In the simulations presented here, changes in tree balance follow a particular type of trajectory: an initial period of wandering within the Yule zone, followed by a “breakout” from Yule-like values leading to the tree becoming sharply imbalanced, rising to a peak imbalance value and then declining back to Yule-like values. We discuss here how this behaviour is explained by the interaction between changes in trait variance, trait-biased speciation, and imposed limits to trait values.

Change in trait variance over time also shows a hump-shaped relationship, but one that is much more regular than that for tree balance (Fig S4a-c, red traces). Peak variance is associated with the maximum span of the range of trait values (see Fig S5c, which corresponds to the peak variance of Fig S4a). Once trait values near the upper limit (for trait-biased speciation probabilities) are attained, the most rapidly-diversifying taxa begin to represent an increasing fraction of all taxa on the tree, resulting in what amounts to a “selective sweep”. Despite diversity-dependence and random background extinction, any speciation that does occur will happen preferentially in taxa with higher speciation probabilities, meaning that clades of more rapidly-diversifying taxa are less likely to go completely extinct in the long run. Because of the limits imposed on trait values (still lower values than those used here would make neither physical nor analytical sense), the distribution of trait variance cannot continue to shift indefinitely. As highly-diversifying taxa at the trait limit become increasingly prevalent, the distribution of trait values becomes increasingly skewed and non-normal (Fig S5c-f), and trait variance declines sharply towards values similar to those at the beginning of the simulation, although the composition of the “population” of taxa has of course changed dramatically.

**Fig S4a-c. Three representative replicates showing offset between maximum trait variance and peak imbalance. Black trace, tree balance; red trace, trait variance; dashed horizontal red line, trait variance = 1.0; dot-dash vertical black line, time of extinction treatment; dashed horizontal red line, time of maximum trait variance; dashed horizontal black line, time of peak imbalance.**

**Fig S5. Histograms showing shift in distribution of trait variance over time. Figs correspond to replicate shown in Fig S3c.**

1. **t = 165, trait variance approximately 1, increasing**
2. **t = 320, trait variance at half-maximum, increasing**
3. **t = 400, maximum variance**
4. **t = 420, half-maximum, descending**
5. **t = 520, variance < 1 but still strong imbalance, descending.**
6. **t = 600, variance at end-simulation**

The trend in tree balance roughly tracks that of trait variance (Fig S4a-c, black traces). First, there is a noticeable lag between the time of peak variance and that of peak imbalance; peak imbalance is attained only *after* trait variance has declined from its peak. Second, while trait variance declines rapidly and smoothly, the decline from peak imbalance is much more irregular and step-like, with the tree often remaining substantially imbalanced even when trait variance has dropped below 1.0. The difference in the behaviour of these two metrics over time is because trait variance is measured without respect to the actual distribution of traits on the phylogeny, whereas tree balance obviously depends critically on that. The presence of even a few more slowly-diversifying relict clades can act to keep imbalance elevated above Yule zone values, even if they contribute very little to the overall calculation of trait variance (see main text Fig 3). Only when these clades have finally gone completely extinct will trait variance actually be sufficiently low for the tree to return to Yule-like values.

In order to further examine this relationship, and to verify that the observed behaviour was not caused by excessively high speciation probabilities, we ran 30 additional simulations of length 1200 ticks in which the lower limit for trait values was first set to 6.0 for the first 800 ticks, so that speciation probabilities could not be more than twice the starting rate. For the final 400 ticks, the lower limit on trait values was then set to 2.0 as it was in the main simulations. If the behaviour in trait variance and tree balance is due to exhaustion of trait/rate variance as limits to trait evolution are encountered, then we should expect to see two distinct peaks in in these metrics, provided there is a long enough time between the shift from the first to second limit on trait values for a complete decline from the first peak to occur. Indeed, this is just what obtains (Fig S6). The peak trait variances attained are smaller than in the main simulations (given the reduced range of trait values that can be realized in each of the two time phases), and the peak imbalances sometimes fail to break far (or at all) out of the Yule zone, but the anticipated behaviour nonetheless emerges. First, when the upper trait limit is encountered and taxa at or close to that value dominate the tree, then again as the lower trait limit comes into effect, allowing for evolution of still more rapidly-diversifying taxa and a temporary renewal of trait/rate variance until taxa at the second limit are realized and sweep the tree. The offset between peak variance and peak imbalance is also seen here; only in 1/30 replicates does peak imbalance occur before peak variance, and only for the first phase. While the time between phases chosen here was entirely arbitrary, it was of sufficient length to demonstrate the occurrence of a single variance peak and Yule zone breakout/decline due to erosion of trait variance once a hard limit is attained, and that this phenomenon can recur if trait/rate variance can be renewed.

**Fig S6. Thirty replicates of two-phase experiments showing double peak in trait variance and offset between trait variance and imbalance peaks for each phase (see Data S1). Each coloured trace is an individual replicate. Upper panel, trait variance; lower panel, tree balance.**
